# Adenine Enrichment at the Fourth CDS Residue in Bacterial Genes Is Consistent with Error Proofing for +1 Frameshifts

**DOI:** 10.1093/molbev/msx223

**Published:** 2017-08-24

**Authors:** Liam Abrahams, Laurence D Hurst

**Affiliations:** 1 Department of Biology and Biochemistry, The Milner Centre for Evolution, University of Bath, Bath, United Kingdom

**Keywords:** frameshift, error mitigation, dual coding, fourth site, translation initiation

## Abstract

Beyond selection for optimal protein functioning, coding sequences (CDSs) are under selection at the RNA and DNA levels. Here, we identify a possible signature of “dual-coding,” namely extensive adenine (*A*) enrichment at bacterial CDS fourth sites. In 99.07% of studied bacterial genomes, fourth site *A* use is greater than expected given genomic *A*-starting codon use. Arguing for nucleotide level selection, *A*-starting serine and arginine second codons are heavily utilized when compared with their non-*A* starting synonyms. Several models have the ability to explain some of this trend. In part, *A*-enrichment likely reduces 5′ mRNA stability, promoting translation initiation. However *T*/*U*, which may also reduce stability, is avoided. Further, +1 frameshifts on the initiating ATG encode a stop codon (TGA) provided *A* is the fourth residue, acting either as a frameshift “catch and destroy” or a frameshift stop and adjust mechanism and hence implicated in translation initiation. Consistent with both, genomes lacking TGA stop codons exhibit weaker fourth site *A*-enrichment. Sequences lacking a Shine–Dalgarno sequence and those without upstream leader genes, that may be more error prone during initiation, have greater utilization of *A*, again suggesting a role in initiation. The frameshift correction model is consistent with the notion that many genomic features are error-mitigation factors and provides the first evidence for site-specific out of frame stop codon selection. We conjecture that the NTG universal start codon may have evolved as a consequence of TGA being a stop codon and the ability of NTGA to rapidly terminate or adjust a ribosome.

## Introduction

A simplistic model of protein-coding gene evolution assumes that amino acid composition is a reflection of selection optimizing the biochemical function of the encoded protein. Consistent with such a model, domains or individual positions critical to protein function are under strong purifying selection (Guo et al. 2004; Furlong and Yang 2008; Gray and Kumar 2011; McFerrin and Stone 2011). Such is the strength of selection on particular amino acids that methods predicting protein domain function from amino acid content are of great utility (Al-Shahib et al. 2007; Sankararaman et al. 2009).

We are becoming increasingly aware of selection pressures beyond those specifying the amino acid sequence acting on coding sequence (CDS) composition. For example, eukaryotic exonic splice enhancers (ESEs) are purine-rich binding-site motifs found at exon ends assisting recruitment of the splicing machinery by regulatory proteins (Blencowe 2000; Graveley 2000; Cartegni et al. 2002; Zhou and Fu 2013). Consequently, codon and amino acid content toward exon ends is biased (Willie and Majewski 2004; Chamary and Hurst 2005a; Parmley and Hurst 2007; Caceres and Hurst 2013) with nonsynonymous and synonymous mutations in ESEs under purifying selection (Fairbrother et al. 2004; Xing and Lee 2005; Carlini and Genut 2006; Parmley et al. 2006; Wu and Hurst 2015). More generally, RNA binding proteins of all flavors impose purifying selection on CDSs (Savisaar and Hurst 2017). There are claims that the CDS is under selection to bind transcription factors (Stergachis et al. 2013), although these are contested (Xing and He 2015; Agoglia and Fraser 2016). Selection might be for avoidance of, rather than selection for, certain motifs, such as intra-CDS Shine–Dalgarno (SD)-like sequences (Diwan and Agashe 2016; Yang et al. 2016), or motifs for RNA binding proteins that bind to introns are avoided within CDSs (Savisaar and Hurst 2017).

A common fingerprint of additional CDS functionality is biased codon usage. Aside from selection for ESEs, codon choice is thought to be affected by, for example, translational selection (Behura and Severson 2011; Doherty and McInerney 2013; Ma et al. 2014), the positioning of nucleosomes (Warnecke et al. 2008; Cohanim and Haran 2009; Prendergast and Semple 2011) and cotranslational protein folding (Zhang et al. 2009; Yu et al. 2015; Buhr et al. 2016). Both RNA and protein structural effects may influence the selection for differential nucleotide content (Chamary and Hurst 2005b; Meyer and Miklós 2005; Shabalina et al. 2006; Gu et al. 2010; Smith et al. 2013; Babbitt et al. 2014). Additionally, intra-CDS microRNA (miRNA) pairing can also impose purifying selection on synonymous mutations in miRNA target sites but, given the span of such binding sites, it is likely they affect nonsynonymous mutations too (Hurst 2006; Forman et al. 2008; Guo et al. 2008; Liu et al. 2015).

The great majority of the above additional levels of information have been identified via hypothesis led approaches (e.g., if ESEs impose selective constraints, we should see ESE-associated synonymous sites conserved at exon ends). An alternative approach is to explore unusual codon or amino acid patterns as strong signals might act as excellent guides to features that are *a priori* important for the operation of cells. Here we highlight one such feature: in bacteria, there is a common bias at CDS fourth sites (i.e., immediately after the initiating codon) for amino acids whose codons start with adenine (*A*). The prevalence of *A*-starting second codons and positive influence on expression has previously been described (Looman et al. 1987; Stenstrom et al. 2001; Zalucki et al. 2007; Zamora-Romo et al. 2007), although these studies were only conducted in *Escherichia coli*. A large-scale multi-genome analysis by Tang et al. (2010) identified a preference for *A* in the first position and *C* in the second position of the second codon, but provided no context as to why the fourth site *A* bias may occur.

We begin by establishing how common bacterial fourth site *A* use is, asking whether it is simply explained by genome GC content influencing codon usage. We establish that the trend remains highly significant after such control in the great majority of bacterial genomes. In some cases, the bias is extraordinarily extreme (over 60% fourth site *A* usage in some genomes). We provide evidence that the fourth site is unusual, even compared with closer nucleotide neighbors. Consistent with strong selection on highly expressed genes, *A* usage is elevated in the most highly expressed genes (although the effect is not dramatic).

Having established that fourth site *A* enrichment is a common and potentially nontrivial feature, we propose and test a number of alternative hypotheses. We start by dismissing some possibilities and then consider three viable models: selection at the protein level requires an *A*-starting codon; RNA level selection minimizes 5′ mRNA domain secondary structures; or that fourth site *A* acts as an immediate trap for +1 frameshifted ribosomes (ATGA becomes TGA on a +1 frameshift). We find that RNA structural selection contributes some of the bias (enrichment is still observed in genomes that don’t use TGA as a stop, but only to the level of enrichment seen downstream), however the frameshift correction model makes for a parsimonious explanation. To the best of our knowledge, this frameshift hypothesis is novel and extends the current understanding of the role of out of frame stop codons, providing the first evidence for site-specific selection of stop codons out of frame. This preference for *A* at the fourth site may, in addition, have become canalized and so feature as part of the start codon recognition mechanism. It is also possible that usage of TGA as a stop codon may also have been related to the evolution of NTG as a start codon.

## Results

### Fourth Site *A* Enrichment Is Common, Sometimes Extreme and Exceptional

#### Controls for Nucleotide Content Confirm a Common and Sometimes Extreme Enrichment of A at CDS Fourth Sites 

**Fig. 1.**
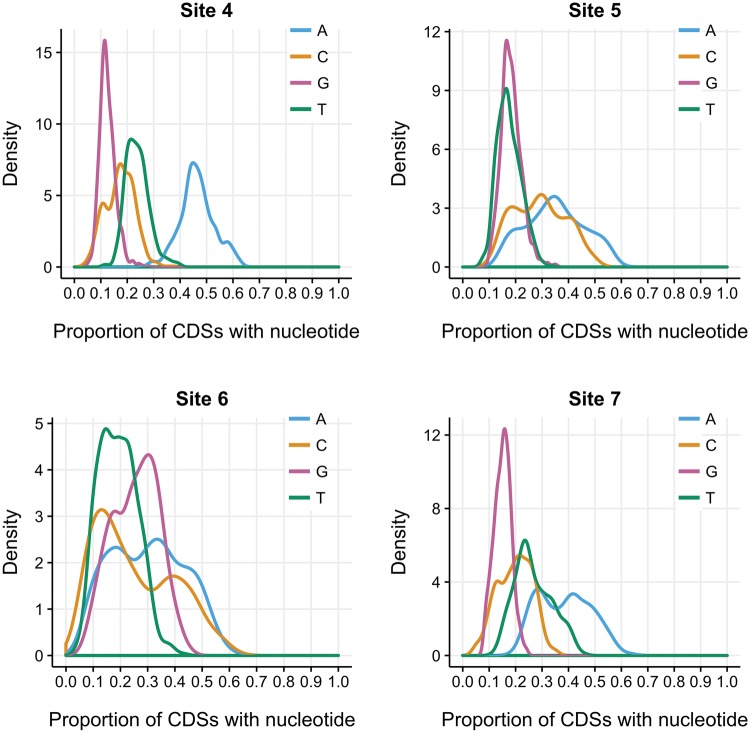
Kernel density plots showing the proportion of coding sequences with each nucleotide (*A, C, G, T*) at coding sequence sites 4, 5, 6, and 7 (site 1 is defined as the first nucleotide of the start codon). Site 4 demonstrates a clear preference for *A* which is not observed at the other sites.

Analysis of bacterial genomes CDSs indicates that in most genomes there is enrichment of fourth site *A* content (fig. 1). The most extreme is *Polaribacter* sp. in which 63.26% of CDSs have *A* at the fourth site. To control for genomic GC effects, we performed a ratio test (see Materials and Methods) comparing the nucleotide usage in the first position of the second codon with nucleotide usage at the first position for all codons in genome. Ratios equal to 1 signify *A*-starting second codons are used proportionately to *A*-starting codons within the genome. We find a remarkable 640/646 genomes (99.07%) have an *A*_4_ ratio significantly >1 (*P *< 0.01, Pearson’s cumulative test statistic [χ^2^], Bonferroni correction). In comparison, 31/646 (4.80%), 3/646 (0.46%), and 55/646 (8.51%) and genomes have *C*_4_, *G*_4_, and *T*_4_ ratios >1, respectively, confirming fourth site enrichment is specific to *A* and not attributable to GC biases. This exceptionalism of the fourth site is further illustrated by the striking reduction in fourth site GC variation (supplementary fig. S1, Supplementary Material online).

#### Fourth Site A Is Conserved 

**Fig. 2.**
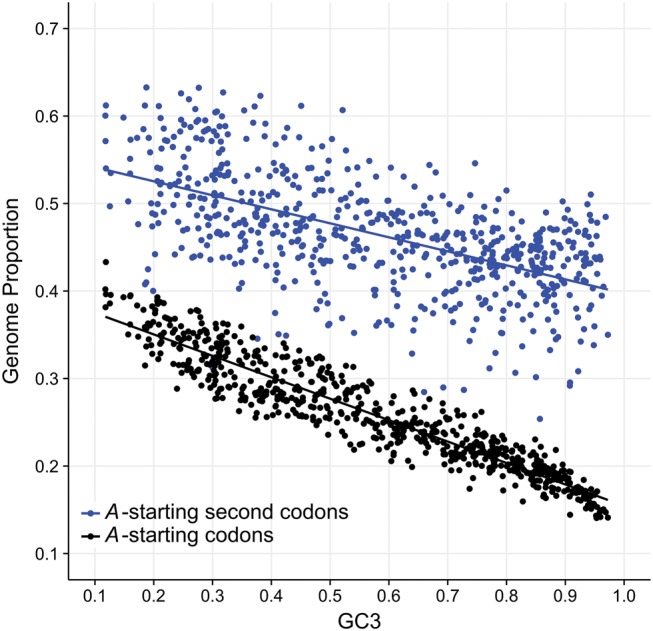
The proportion of coding sequences with fourth site *A* is maintained above the proportion of *A*-starting codons as GC content increases. The regression coefficient for all *A*-starting codons is significantly greater than for *A*-starting second codons (*P *= 7.056 × 10^−19^, *Z* = 8.874, two-tailed *Z*-test of equivalency), suggesting enrichment of *A* at the fourth site becomes stronger with increasing GC content.

Genomes with high “silent” GC content (GC3) tend to more readily employ the amino acids with GC rich nonsynonymous sites (Warnecke et al. 2010). This shift in amino acid content we term GC “pressure.” If the usage of *A* at fourth sites is functionally relevant we would expect its usage to be more resilient to GC pressure than for *A*-starting codons within the genome. Comparing genomic GC3 with both the proportion of *A*-starting second codons and all *A*-starting codons (fig. 2), we observe that the regression coefficient for all *A*-starting codons (−0.245) is significantly more negative than for *A*-starting second codons (−0.160) (*P *= 7.056 × 10^−19^, *Z* = 8.874, two-tailed *Z*-test of equivalency) and thus *A* at the fourth site is more resilient to genomic GC pressures.

**Fig. 3.**
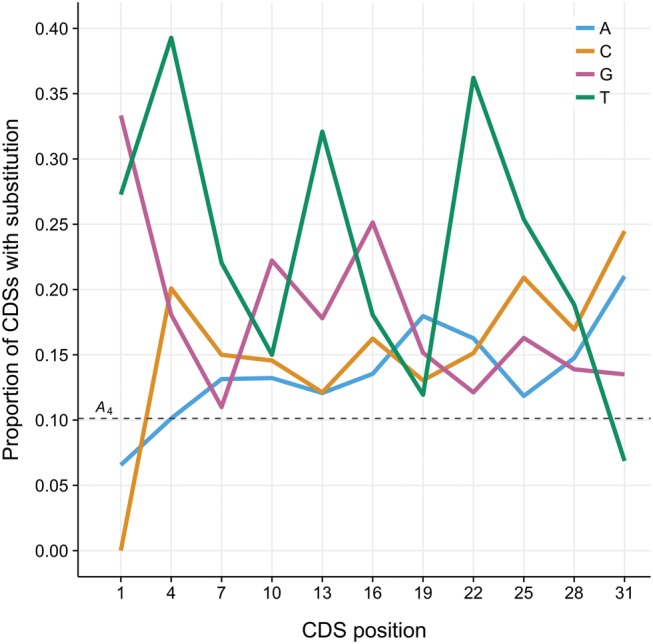
The proportion of *Shigella flexneri* orthologs with a substitution of each nucleotide at the first position of codons from *Escherichia coli*. The proportion of sequences with a substitution from *A* at site 4 is displayed with the dotted line. Position 1 of the first codon demonstrates minimal variation away from an *A*-genotype confirming the preference for an ATG start codons. Substitutions from an *A*-genotype are reduced across the sites when compared with other nucleotides. The proportion of coding sequences with a change from *A* in codon 2 is significantly lower than neighboring codons (*P* < 0.001, one-sample *T*-test), suggesting fourth site *A* is under strong selection.

Further evidence of functionality arises from analysis of the conservation of fourth site *A* between *E. coli* and *Shigella flexneri*. *E. coli* and *Shigella* spp. are closely related (Pupo et al. 2000; Zuo et al. 2013), demonstrating high nucleotide similarity between species (Goris et al. 2007). *Shigella* spp. undergo accelerated gene loss when compared with *E. coli*, in part explained by weakened purifying selection associated with reduced effective population size (*N_e_*) (Hershberg et al. 2007; Balbi et al. 2009). Thus, if there is selection at the fourth site, by focusing on *E. coli* residues we can ask whether fourth site *A* is particularly resilient to substitution to an alternative nucleotide under weaker purifying selection by comparing with a lower *N_e_* comparator for which purifying selection, as a result of reduced *N_e_*, will be less effective in purging deleterious substitutions. If the fourth site is under particularly strong selection, we expect substitutions at the fourth site to be reduced when compared with other sites. We find the proportion of CDSs differing from *A* at the fourth site in *S. flexneri* is lower than for other nucleotides (fig. 3). This result assumes the *E. coli* state to be more reflective of the ancestral state, particularly as the low *N_e_* genome is expected to have a higher rate of change. Although other first codon positions demonstrate a relative reduction away from an *A*-genotype when compared with other nucleotides, loss of *A* in the fourth position is significantly reduced compared with downstream positions (*P* < 0.001, one-sample *T*-test). This lack of change specific to the fourth site *A* genotype is indicative of purifying selection at the fourth site.

#### More Highly Expressed Genes Have Higher Fourth Site A Content 

Selectively relevant features are often more pronounced in highly expressed genes (Urrutia and Hurst 2003; Doherty and McInerney 2013). To assay expression level, we consider the Codon Adaptation Index as a surrogate. For genomes in which suitable annotations were available, we compared the mean CAI for genes with and without fourth site *A* (N.B. this paired test controls for residual effects such as intergenome GC variation). We find a significantly higher CAI for genes with fourth site *A* (*P* = 1.042 × 10^−12^, *N* = 232, paired Wilcoxon rank-sum test), although the mean CAI value for CDSs with fourth site *A* (0.586 ± 0.088, *N* = 232) is only slightly greater (0.582 ± 0.088, *N* = 232) than for those without. Performing the test in the opposite direction, we find a significant increase (*P *= 0.034, Wilcoxon rank-sum test) in the proportion of CDSs with fourth site *A* in the highly expressed genes (0.457 ± 0.07, *N* = 232) compared with those less expressed (0.454 ± 0.082, *N* = 232).

The above result is most pronounced in high GC genomes. Genomes with extreme GC compositions demonstrate a reduced range of mean CAI values (supplementary fig. S2, Supplementary Material online) (Botzman and Margalit (2011) with codon usage in many CDSs similar to that for the ribosomal proteins. Repeating the same analyses for just 30 genomes with 20% ≤ GC3 ≥ 90% (supplementary fig. S2, red, Supplementary Material online) (reducing the mean CAI range to 0.576–0.743) we find mean CAI values for CDSs with fourth site *A* significantly higher (*P* = 1.486 × 10^−6^, paired *T*-test, *N* = 30) but again the difference in mean CAI in CDSs using *A* (mean CAI = 0.661 ± 0.034, *N* = 30) and non-*A* (mean CAI = 0.650 ± 0.036, *N* = 30) is small. For the GC-rich genomes, we find a significant difference in mean CAI (*P* = 4.451 × 10^−10^, paired *T*-test, *N* = 18) for CDSs using fourth site *A* (mean CAI = 0.581 ± 0.089, *N* = 18) when compared with those that do not *A* (mean CAI = 0.581 ± 0.089, *N* = 18). However, for AT-rich genomes mean CAI values are not significantly different between those using fourth site *A* and those not (*P* = 0.243, paired *T*-test, *N* = 12). These results suggest that fourth site *A* is more commonly utilized in highly expressed genes, albeit to a small degree, and even maintained under extreme GC restrictions. However, when conditions are inherently conducive to incorporating an *A*-starting second codon, we expect *A*-starting second codons to be used regardless of alternative selection pressures and therefore any enrichment signal is harder to detect.

### Three Models to Explain Selection for Fourth Site *A* Content

Our results thus far all support the exceptionalism of the fourth site. Why might this be? The 5′ CDS is known to have distinct selection pressures to those acting on the remainder of the CDS. Although 5′ ends are enriched with nonoptimal codons (Tuller, Carmi et al. 2010; Pechmann and Frydman 2013; Tuller and Zur 2015), Bentele et al. (2013) have demonstrated that in bacteria selection favors codons that reduce mRNA folding around the translation start, regardless of whether these codons are frequent or rare. Notably, when a nonoptimal codon is GC-rich, they find preferences for optimal AT-rich codons. Thus, the trend is not explained by selection for nonoptimality (also concluded by Eyre-Walker and Bulmer 1993) but AT-content and therefore we do not consider this a selection pressure. An alternative explanation could be the presence of overlapping genes: a CDS employing the TGA stop codon overlapping a downstream CDS by four nucleotides will result in an *A* nucleotide in the fourth position of the subsequent CDS. However, after removing 165,357/2,173,531 (7.61%) CDSs with these four site overlaps, 635/646 (98.30%) genomes achieve an *A*_4_ ratio >1 (*P *< 0.01, Pearson’s cumulative test statistic (χ^2^), Bonferroni correction) and therefore overlaps cannot account for the fourth site enrichment. Are there alternative explanations? We propose three possible models, which we proceed to test.

#### The Amino Acid Preference Model 

Certain amino acids (lysine, serine) have been shown to be favored immediately following the start codon in both prokaryotes and eukaryotes (Shemesh et al. 2010) and evidence suggests that these amino acids may provide important functional roles (Stenstrom et al. 2001). Furthermore, Tats et al. (2006) and Bivona et al. (2010) note particular amino acids (alanine, cysteine, proline, serine, threonine, and lysine) may be used more frequently in the second position in highly expressed genes. These observations may be attributed to involvement of the second amino acid in posttranslational modifications. N-terminal methionine excision (NME) only occurs when the second amino acid is glycine, alanine, serine, threonine, cysteine, proline, or valine—amino acids with small side chains (Liao et al. 2004; Frottin et al. 2006; Ouidir et al. 2015). The second amino acid is implicated in the N-end rule pathway (overview in Tasaki et al. 2012), targeting proteins for degradation (Bachmair et al. 1986; Tobias et al. 1991) with the main determinants the amino acids not involved in NME (Varshavsky 2011). Signaling proteins requiring the inclusion of specific concentrations of hydrophobic amino acids (Ng et al. 1996) may also contribute to amino acid bias. A variety of protein-level selection pressures may therefore be acting upon the second amino acid.

If enrichment reflects protein-level selection on the second amino acid, we expect no difference in the use of *A*/non-*A* starting six fold degenerate amino acids as it is simply the amino acid, not the underlying nucleotide, that is important. We also expect other non-*A* starting amino acids to be favored given post-translational modification requirements.

#### The RNA Stability Model 

Reducing secondary RNA structures in 5′ mRNA domains enhances the ability of the mRNA to interact efficiently with ribosomes and promotes translation efficiency (de Smit and van Duin 1990; Tuller, Waldman et al. 2010; Scharff et al. 2011). There indeed exists a relationship between 5′ mRNA folding strength and protein expression levels in prokaryotes and eukaryotes (Kudla et al. 2009; Li, Zheng, Ryvkin et al. 2012; Li, Zheng, Vandivier et al. 2012; Bentele et al. 2013; Goodman et al. 2013; Shah et al. 2013; Vandivier et al. 2013). Minimising the presence of these secondary structures, for example hairpin loops, by adopting destabilizing AT-rich 5′ domains (Qing et al. 2003; Kudla et al. 2009; Gu et al. 2010; Bentele et al. 2013; Goodman et al. 2013) could therefore promote more efficient translation by facilitating mRNA-ribosome interactions. Several studies have experimentally identified second codon AT preference promoting faster translation initiation (Zalucki et al. 2007) and correlating positively with expression levels (Stenstrom et al. 2001).

If reducing RNA stability can explain the fourth site *A* enrichment, we would expect enrichment at the fourth site to not be unique, but representative of neighboring codons in the 5′ mRNA binding domain. For instance, we would expect no significant difference between the fourth, seventh and tenth sites or between synonymous sites in these codons. Furthermore, if there is uniquely selection for increased AT-content to destabilize the RNA, we also expect to see a localized *T* enrichment.

#### The Frameshift Correction Model 

**Fig. 4.**
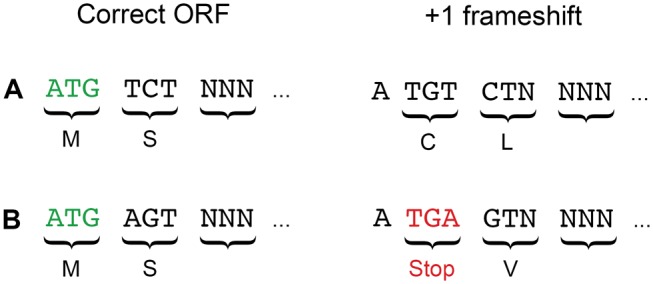
A schematic representation of the frameshift correction model. Both CDSs encode methionine followed by serine and have identical GC content. However, following a +1 frameshift sequence A encodes a cysteine followed by a leucine, whereas translation of sequence B is immediately terminated by the presence of an out of frame TGA stop codon.

Consider a CDS that starts NTGA, with *A* at the fourth site. Following a + 1 frameshift, this sequence becomes the TGA stop codon, immediately terminating or realigning translation and preventing the ribosome continuing on a + 1 reading frame (overview in fig. 4). We define this as the frameshift correction model, providing a novel and site-specific case of out of frame stop codons more generally.

This model presumes a + 1 frameshift is deleterious. Whilst viruses (Su et al. 2005; Melian et al. 2014), prokaryotes (Tsuchihashi and Kornberg 1990; Gupta et al. 2013) and eukaryotes (Wills et al. 2006; Belew et al. 2014) (reviewed in Caliskan et al. 2015) do employ frameshifting to encode multiple proteins from one mRNA strand (e.g., the *gag-pol* gene; Jacks et al. 1988), many ribosomal frameshifts are errors. Ribosomes leaving the correct reading frame and synthesizing proteins that were never “intended” are likely to incur cellular costs (Warnecke et al. 2010). For example, reduced ribosomal capability can be rate limiting for growth (Shachrai et al. 2010), whilst important cellular resources (tRNAs, amino acids) are misinvested. Furthermore, incorrectly folded mistranslated proteins may have an adverse effect on cellular interactions or form toxic aggregates (Tank and True 2009). The possible evolutionary advantage of capturing these frameshifts is conjectured to be reflected by an overrepresentation of out of frame stop codons, termed the “ambush hypothesis” (Seligmann and Pollock 2004; Singh and Pardasani 2009; Tse et al. 2010), although the frequency with which codons that form out of frame stops are used is largely predictable from the underlying GC pressure (Morgens et al. 2013). Alternatively, selection to reduce costs in genomes where frameshifting is most deleterious (notably GC rich ones) can explain the richer tRNA repertoire found in such genomes (Warnecke et al. 2010).

This +1 frameshift correction mechanism requires a NTG start codon. Prokaryotes are known to use a variety of non-ATG start codons with varying efficiencies (O'Donnell and Janssen 2001; Panicker et al. 2015), however 99.84% of CDSs within genomes in this study use a NTG start codon (supplementary table S1, Supplementary Material online), with ATG, GTG, and TTG the most highly represented (80.97%, 13.02%, and 5.72%, respectively). If this frameshift correction model can help to explain observed fourth site *A* enrichment, we can expect weaker enrichment in genomes that do not use TGA as a stop codon. Furthermore, the distance to the next +1 stop codon may be greater as initial frameshifts are captured immediately.

### Testing the Models

#### The Amino Acid Preference Model Cannot Explain A-Starting Amino Acid Biases in the Second Peptide Position

##### A-Starting Codons Are Preferred Even If There Are Synonymous Alternatives

The structure of the genetic code provisions us with a natural test. Six-fold degenerates serine, leucine, and arginine are encoded by synonymous codons in two codon blocks, in which the first position nucleotide varies. *A*-starting codons for serine (S_*A*_) and arginine (R_*A*_) account for one third of the total codons available. Thus, if there is an amino acid level selection we expect to see mostly *T*-starting serine (S_*T*_) and *C*-starting arginine (R_*C*_).

Serine is especially informative. Assuming selection is primarily for the amino acid content of serine, we expect to see no difference between enrichment of both coding blocks as both maintain AT content destabilizing the 5′ mRNA domain. Whilst both S_*A*_ and S_*T*_ are more frequent in the second position than expected given genome amino acid usage (*P* < 0.001, Pearson’s cumulative test statistic [χ^2^]), the mean deviation within genomes from the expected number of CDSs utilizing serine as the second amino acid is greater for *A*-starting (mean observed—expected #x0003D; 170.186) than *T*-starting serine (mean observed—expected = 70.774). In an unbiased genome, we would expect, all else being equal, the ratio of S_*A*_:S_*T*_ to be 1:2. For all amino acids in the genome, we find the mean S_*A*_:mean S_*T*_ ratio equal to 1:1.762 (*N* = 646), however for the second amino acid this ratio is 1:0.821, again indicating a strong *A*-starting second amino acid bias. Using genome serine use as our null, we find a significant increase of *A*-starting serine at the second site (*P* < 0.001, Pearson’s cumulative test statistic [χ^2^]). Furthermore, *A*-starting serine enrichment ratios (mean ratio = 3.429 ± 1.839, *N* = 646) are significantly greater (*P* < 2.2 × 10^−16^, paired Wilcoxon rank-sum test) than for *T*-starting serine (mean ratio = 1.535 ± 0.526, *N* = 646). It is apparent that there is a distinct overrepresentation of *A*-starting serine in the second site, indicating selection specific to the *A*-nucleotide.

A comparable analysis for *A*/*C*-starting arginine amino acids is slightly less discriminatory as *C*-starting arginine does not maintain the AT-content. Given genome amino acid usage, we find *A*-starting arginine overrepresented in the second position (*P* < 0.001, Pearson’s cumulative test statistic [χ^2^]; mean observed – expected = 49.107) with *C*-starting arginine underrepresented (*P* < 0.001, Pearson’s cumulative test statistic [χ^2^]; mean observed – expected = −26.319). A ratio of 1:4.390 for genome mean R_*A*_:mean R_*C*_ (*N* = 646) use demonstrates greater dependence on *C-*starting arginine within CDSs, however a second amino acid ratio of 1:1.565 highlights the greater dependence on *A*-starting arginine at the second site. With genome arginine use as the null, we find a significant increase of *A*-starting arginine at the second site (*P* < 0.001, Pearson’s cumulative test statistic [χ^2^]). *A*-starting arginine (mean ratio = 3.492 ± 2.338, *N* = 646) enrichment ratios are significantly greater (*P* < 2.2 × 10^−16^, paired Wilcoxon rank-sum test) than for *C*-starting arginine (mean ratio = 0.892 ± 0.384, *N* = 646).

Evidently, *A*-starting synonyms of both serine and arginine are favored at the second position indicating selection is stronger for the *A* nucleotide in the first codon position and that selection is not at, or strongest at, the protein level.

##### No Individual Amino Acids Are Uniquely Preferred in the Second Peptide Position

We also consider whether enrichment reflects selection for specific *A*-starting amino acids in the second position, which could be expected were we witnessing selection at the peptide level. Conversely, if selection were at the nucleotide level we expect multiple amino acids with *A-*starting codons to be over-represented so long as they facilitate posttranslational modifications.

**Fig. 5.**
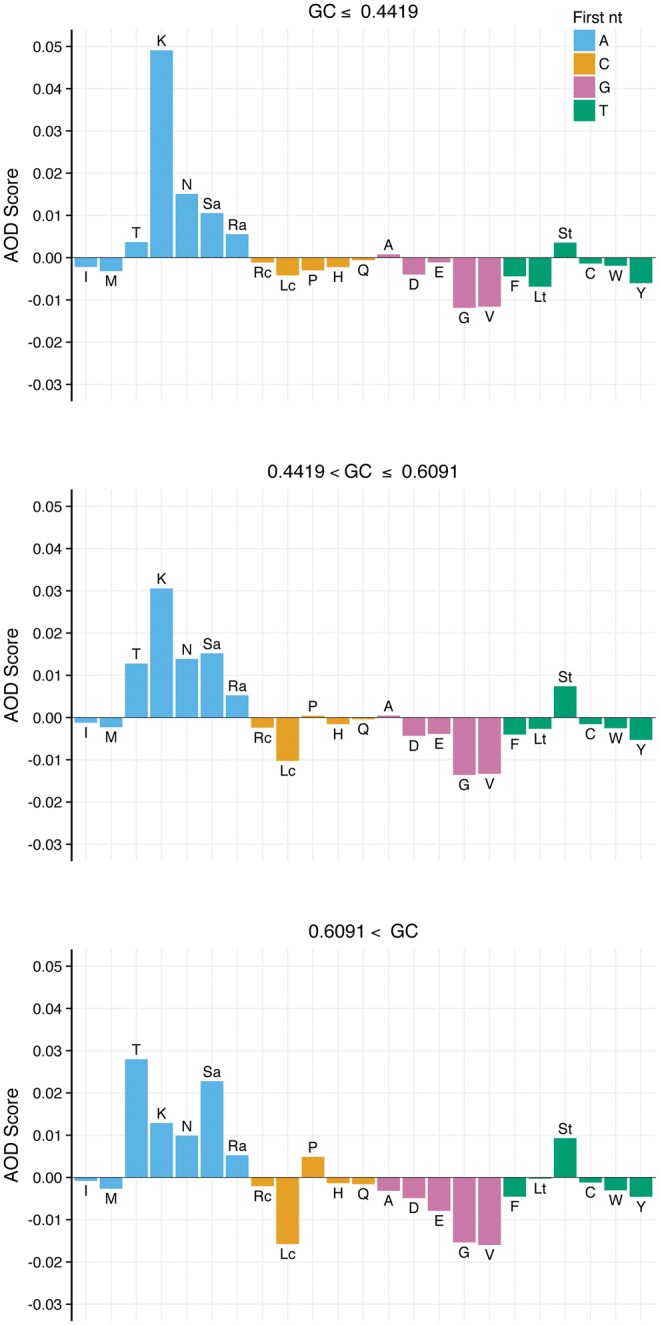
Average of difference (AOD) scores for each amino acid, demonstrating enrichment or avoidance of each amino acid in the second peptide position when compared with amino acid use within the transcriptome. Genomes are grouped by GC content into three equal sizes grouping in order to minimize GC biases on amino acid choice (lysine for example, encoded by AAA and AAG, is expected to be used more frequently in GC-poor genomes). Amino acids encoded by two coding blocks are defined using the first nucleotide in the codon, for example, *A*-starting serine is denoted Sa. A preference for *A*-starting amino acids except methionine and isoleucine, regardless of genome GC content, is observed.

To determine second position amino acid preferences, we calculated average of difference (AOD) scores (see Tang et al. 2010). AOD scores distinguish whether there is a preference and enrichment of particular amino acids in the second position when compared with the whole transcriptome. In a similar manner to Tang et al. (2010), genomes were categorized into three equal groupings of low GC content (GC ≤ 44.19%), medium GC content (44.19% < GC ≤ 60.91%) and high GC content (60.91% < GC) to limit genomic GC effects. Each amino acid encoded for by *A*-starting codons is preferred at the second position regardless of genome GC content, except for methionine and isoleucine (fig. 5). Avoidance of methionine–methionine cannot be attributed to general avoidance of methionine pairs as they are found more frequently than expected given genome methionine usage (*P* < 0.001, Pearson’s cumulative test statistic [χ^2^]). However, as methionine in the second position doesn’t facilitate NME, the avoidance may be related to the cleaving mechanism. Conversely, genome methionine-isoleucine pairs are less frequent than expected (*P* < 0.001, Pearson’s cumulative test statistic [χ^2^]) and therefore a general avoidance of methionine-isoleucine pairs may provide some explanation for second site avoidance.

Bonissone et al. (2013) propose that the primary role of NME is to expose serine and alanine rather than other NME substrates, possibly explaining why *T*-starting serine is the only non *A*-starting amino acid universally preferred across GC groupings. Regarding posttranslational modifications this makes sense—for CDSs with non-*A* starting second amino acids we still expect to see an amino acid capable of participating in NME. As we previously describe, both serine blocks are preferred, although *A*-starting serine amino acids are favored. The ability to facilitate NME may explain weak proline and alanine preferences and the preference for threonine and serine(*T*) in GC-rich genomes where *A*-starting codon usage is limited.

If selection is primarily for amino acid functionality, non-*A* starting amino acids involved in modifications should be preferred. This is not the case. Primary N-end rule pathway residues (leucine, phenylalanine, tyrosine, and tryptophan) recognized directly by the bacterial N-recognin ClpS (Dougan et al. 2012) are avoided. For secondary residues (methionine, lysine, and arginine) signaling for the attachment of a primary residue by leucyl/phenylalanyl-tRNA-protein transferase (LFTR) (Dougan et al. 2012), methionine is avoided with only *A*-starting amino acids preferred (avoidance of *C*-starting arginine). Conversely, if selection is at the protein level *A-*starting amino acids not involved in cleavage should be avoided. This is also not seen; *A*-starting asparagine is preferred but does not feature in either posttranslational modification pathway. More generally, the use of *A-*starting amino acids not involved in either pathway (lysine, asparagine, arginine) further suggests selection is operating on underlying nucleotide content.

#### 5′ RNA Structure Requirements Cannot Fully Account for Fourth Site *A* Enrichment

The amino acid analysis suggests that selection is not for amino acids themselves but for *A*-starting codons (provided protein function is not overly compromised). If the selective constraint is to reduce 5′ mRNA stability, we also expect a degree of *T* enrichment within this domain. This prediction comes with the caveat that *G*:*U* noncanonical pairing is possible and could act to increase RNA stability (Varani and McClain 2000). *T*_4_ ratios are significantly reduced compared with *A*_4_ ratios for each genome (*P* < 2.2 × 10^−16^, paired Wilcoxon rank-sum test. Indeed the mean *T*_4_ ratio is 0.796 ± 0.156 (*N* = 646) whereas the mean *A*_4_ ratio is 1.873 ± 0.375 (*N* = 646), indicating that the effect is relatively *A* specific.

If selection is acting to increase *A* content, we expect little difference between *A* enrichment of the second codon and contiguous codons at both synonymous and nonsynonymous sites. GC variability at synonymous sites is more extreme than at other positions (Muto and Osawa 1987), allowing the possibility of regulation of local GC content independently of amino acid requirements (Babbitt et al. 2014). We therefore predict that if there is selection for *A-*rich codons in the 5′ domain, GC content at synonymous sites should be more independent of genome GC content than codons downstream. Results indicate this is the case (supplementary fig. S3, Supplementary Material online).

This resilience to GC pressure in the 5′ mRNA domain is suggestive of alternative selection pressures acting to determine synonymous site composition. If selection is being driven by RNA stability requirements, we might expect to observe selection on *A* content at all synonymous sites immediately 3′ of the start codon, but with little difference to synonymous sites of immediate codon neighbors. The mean *A*_6_ ratio (1.954 ± 0.802, *N* = 646) confirms *A*-enrichment. Comparisons between *A*_6_ ratios with *A*_9_ and *A*_12_ ratios (in codons 4 and 5) show weakly significant *A* content variation at these synonymous sites (*P *= 0.041, KruskalWallis rank-sum test), however pairwise comparisons between *A* ratios indicate the second codon is not significantly different in terms of synonymous *A* enrichment (*A*_6_–*A*_9_: *P *= 0.973, *A*_6_–A_12_: *P *= 0.057, *A*_9_–*A*_12_: *P* = 0.096, pairwise Tukey–Kramer tests). Extending the analysis to the fifth codon, we find synonymous site *A* enrichment significantly decreases (*P *< 0.01, Kruskal–Wallis rank-sum test; *A*_6_–*A*_15_: *P *= 1.2 × 10^−8^, *A*_9_–A_15_: *P *= 4.2 × 10^−8^, *A*_12_–*A*_15_: *P* = 0.001, pairwise Tukey–Kramer tests), consistent with stronger selection toward 5′ ends. Enrichment is therefore considered comparable for codons two, three, and four.

**Fig. 6.**
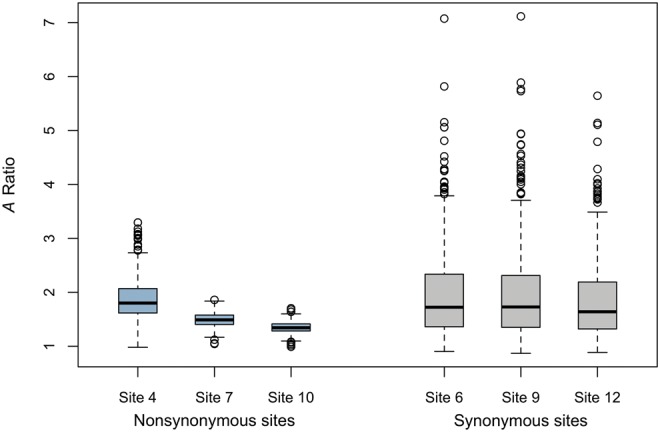
Comparisons between *A* enrichment ratios for synonymous and nonsynonymous sites in codons 2–4. Enrichment ratios compare the use of *A* at each site with at comparable positions for all codons in the transcriptome (i.e., site 4 is compared with the first positions of all codons, site 5 is compared with the second positions of all codons and site 6 is compared with the third positions of all codons). Unlike synonymous sites in neighboring codons that display similar *A* enrichment ratio distributions, we observer greater variation in *A* enrichment ratios for the fourth site in comparison with the more tightly controlled ratios for sites 7 and 10. Enrichment ratios at the fourth site are significantly increased when compared with sites 7 and 10.

But is there a unique enrichment specific to the fourth site? If selection on the fourth site is solely for RNA stability, we expect similar *A-*ratios between the nonsynonymous sites of these neighboring codons, as with synonymous sites. In contrast, we find that *A*_4_ is elevated (fig. 6). There are significant differences between the *A-*ratios at the nonsynonymous sites (sites 4, 7, and 10) (*P* < 2.2 × 10^−16^, log-transformed *A-*ratios, Kruskal–Wallis rank-sum test), with pairwise comparisons suggesting enrichment at each site is significantly different (*P* < 2.2 × 10^−16^, pairwise Tukey–Kramer tests). We find the mean *A*_4_ enrichment (1.873 ± 0.375, *N* = 646) greater than *A*_7_ (1.488 ± 0.129, *N* = 646) and *A*_10_ (1.344 ± 0.105, *N* = 646).

These results highlight that despite AT requirements in the initial 5′ mRNA domain, the fourth site exhibits significant enrichment not observed at other nonsynonymous sites, a trend not seen for synonymous sites. We therefore cannot attribute the increased fourth site *A* content solely to RNA stability selection.

#### The Frameshift Correction Model Is a Parsimonious Explanation

##### The Frameshift Correction Model Predicts Weaker Enrichment at the Fourth Site in Genomes Not Using the TGA Stop Codon

The use of a NTG start codon dictates that under the frameshift model, the stop codon must be the TGA stop codon. If the frameshift model can best explain the enrichment observed, we would expect enrichment at synonymous sites in genomes not using TGA to only occur at levels similar to those in codons 3 and 4 due to 5′ RNA stability constraints.

Five of the 651 genomes within this study (*S. mirum*, *M. gallisepticum*, *M. florum*, *U. parvum*, and the Synthetic construct designed and chemically synthesized from *M. genitalium*; Gibson et al. 2008) use this alternative genetic code (NCBI translation table 4). *A*_4_ ratios demonstrate an enrichment of *A* (1.277, 1.443, 1.548, 1.362, and 1.099, respectively), but, importantly, are significantly lower than the *A*_4_ ratios for genomes using the standard genetic code (*P *< 0.01, Wilcoxon rank-sum test). After removing the Synthetic construct from the analysis, the difference remains significant (*P *= 0.004, Wilcoxon rank-sum test). Furthermore, the *A*_4_, *A*_7_, and *A*_10_ ratios for these genomes exhibit no significant difference between them (*P *= 0.368, Kruskal–Wallis rank-sum test). *A*_4_ ratios are also not significantly different to the *A*_7_ ratios of the third codon (*P* = 0.053, Welch two sample *T*-test) or *A*_10_ ratios for the fourth codon (*P *= 0.835, Welch two sample *T*-test) in genomes using the standard genetic code.

Might the lower *A*_4_ ratio of genomes not using TGA reflect their high AT content more generally? In order to control for GC content, we performed a loess regression between total genomic GC content and *A*_4_ enrichment ratios and compared the residuals for the two different translation tables. In this case, we find no significant difference between the enrichment ratios (*P *= 0.234, Kruskal–Wallis rank sum test). We note however, that the mean residual for the translation table 4 genomes (−0.103) is lower than for the genomes using the standard genetic code (−0.001) although not significant. This is however limited by the small sample size for table 4 genomes (5 genomes). If we include all table 4 genomes from the original data set (*N* = 94), although we introduce some phylogenetic nonindependence, we find the difference highly significant (*P *< 0.001, Kruskal–Wallis rank sum test) (supplementary fig. S4, Supplementary Material online). The mean residual for table 4 genomes is again negative and lower (−0.070) than for those using the standard genetic code (0.006). Supplementary figure S4*A*, Supplementary Material online, suggests that table 4 genomes may fall into two categories: those that have greatly reduced enrichment and those that are similar to genomes using the standard genetic code. This may result from phylogenetic nonindependence introduced when increasing the data set with the majority of genomes being *Mycoplasmas* (75/94; 79.79%). However Supplementary figure S4*C*, Supplementary Material online, suggests *Mycoplasma* residuals are varied. As these genomes are *AT*-rich, is it highly likely these genomes would utilize *A*-starting second codons regardless of fourth site selection, therefore the fact that there is reduced use in 57/94 (60.64%) genomes is suggestive of a difference in these table 4 genomes. Thus, these observations accord with a model in which the absence of TGA as a stop codon relaxes selection for especially high *A*_4_ content. The remaining *A* excess seen can be accounted for in terms of selection for decreased 5′ mRNA stability (as also observed for *A*_7_ and *A*_10_). Assuming high AT content reflects weaker selection against a GC to AT mutation bias, the above results also suggest that the lower *A*_4_ ratios in table 4 genomes cannot be owing to weakened purifying selection (assuming AT content is a proxy for *N*_e_).

##### The Distance to the Next +1 Frameshift Stop Codon Is Greater for Genes with Fourth Site A

The excess of *A* at site four is consistent with preventing the ribosome initiating on the wrong reading frame. If the ribosome begins translation on an incorrect reading frame and is abruptly terminated, there is less demand for another local +1 stop codon (assuming selection for ambush codons). We therefore expect that the distance to the next +1 stop codon in genes with fourth site *A* is greater than those without. As the three standard stop codons are AT-rich (TAA, TAG, and TGA), we find a strong positive correlation between GC content and the mean nucleotide distance to the next +1 stop codon (ρ = 0.966, *P* < 2.2 × 10^−16^, Spearman’s rank correlation) (supplementary fig. S5, Supplementary Material online). We therefore make within-genome comparisons as we can expect GC content to equally influence distances in CDSs with and without fourth site *A.*

The mean distance to a +1 stop codon is significantly greater in genes with fourth site A (*P* < 2.2 × 10^−16^, paired Wilcoxon rank-sum test) but not for genomes not using the standard genetic code (*P* = 0.461, paired *T*-test). The presence of an immediate frameshift correction mechanism therefore appears to influence location of further downstream out of frame stop codons. The mean of mean genome distances shifts from 68.583 ± 37.091 (*N* = 646) nucleotides for genes without fourth site *A* to 72.533 ± 42.376 (*N* = 646) nucleotides in the presence of fourth site *A* with distances varying greatly between genomes. We observe increased distances to the second +1 stop codon from a mean of 141.334 ± 73.537 (*N* = 646) nucleotides without fourth site *A* to 144.718 ± 78.656 (*N* = 646) nucleotides (*P* < 2.2 × 10^−16^, paired Wilcoxon rank-sum test) and the third +1 stop codon from 212.226 ± 105.601 (*N* = 646) nucleotides without fourth site *A* to 215.238 ± 110.525 (*N* = 646) nucleotides (*P* = 1.571 × 10^−13^, paired Wilcoxon rank-sum test). In effect, the incorporation of an immediate +1 stop codon appears to subtly shift the sequence of frameshift capture codons downstream. Although these distances are highly variable (the effects of GC content varying between genomes), by comparing samples from within each genome we limit the effects of this variability. The preservation of *A*_4_ under increased GC pressure (fig. 2) is consistent with stronger selection in GC rich genomes for *A*_4_ preservation given the greater distance to the next +1 stop is likely to incur a greater cost.

## Discussion

### *A*_4_ Content as Another Residue for Error Correction?

We have identified a series of variables that go some way to explaining the enrichment of *A* at fourth sites. For CDSs with upstream SD sequences, we find reduced fourth site *A* use (supplementary result 1, Supplementary Material online), consistent with the notion that SD sequences reduce the error rate at translation initiation when compared with genes lacking ribosome recruitment and initiation signals (Di Giacco et al. 2008)*.* The presence of leader genes synthesizing nonfunctional peptides also go some way to explaining why sequences may lack fourth site *A* (supplementary result 2, Supplementary Material online). A multivariate model using genome *A*-starting codon use, 5′ *A* enrichment, leader gene use and the translation table explains over 50% of the variation in genome fourth site *A* use (supplementary result 3, Supplementary Material online). Given the validity of the frameshift model, we note that such a model might go some way to explain why start codons are in fact of the form NTG. We speculate that in early evolution there may have been coevolution of stop codon usage (we assume TGA to be ancestral) and choice of NTG codons as initiators prior to further dual-coding signals evolving in order to provide more stringent initiation pathways. If so, this provides, to the best of our knowledge, the first explanation as to why start codons are typically NTG and methionine.

The validity of the frameshift model is especially noteworthy given many dual coding signals relate to the control of errors (reviewed in Drummond and Wilke [2009] and Warnecke and Hurst [2011]). For example, splice control by ESEs may be considered as a control of missplicing errors (Dewey et al. 2006; Caceres and Hurst 2013; Wu and Hurst 2015) as ESEs are most abundant near longer introns where splicing error is most common. Selection to avoid amino acid misincorporation (Archetti 2004, 2006; Drummond et al. 2005; Stoletzki and Eyre-Walker 2007; Gilchrist et al. 2009) or codons in close mutational proximity to stop codons where nonsense mediated decay (NMD) cannot detect transcriptional errors (Cusack et al. 2011) may constrain codon choice. The presence of stop codons within introns appears to be NMD-mediated mechanism to catch splice errors (He et al. 1993; Jaillon et al. 2008; Farlow et al. 2010; Mekouar et al. 2010). This suggests a general theme coupling dual coding with error mitigation.

### Is *A*_4_ Enrichment Involved in Translation Initiation?

The notion that CDSs might incorporate +1 stop codons favored by selection is not new. Indeed, it has been proposed that the genetic code evolved such that it has the ability to encode frameshift traps (Itzkovitz and Alon 2007). The ambush hypothesis (Seligmann and Pollock 2004) proposes that there is an excess of out of frame stops and that coding sequences frequently use and are under selection for codons that have the potential to form out of stop codons (Seligmann and Pollock 2004; Singh and Pardasani 2009; Tse et al. 2010). However, the biases toward codons contributing to out of frame stops seems largely predictable from the underlying GC pressure (Morgens et al. 2013) with the ambush hypothesis not strictly observed at the gene level (Bertrand et al. 2015). The observation that the usage of *A* at the fourth site is significantly increased in genomes employing the TGA stop is perhaps the first evidence that selection does favor, at least at one specific site, out of frame stop codons.

Why might the fourth site be unusual and warrant a frameshift trap? We suggest that this might relate to the process of translation initiation itself. The results are consistent with a frameshift correction model, however the dynamics in which the ribosome may find itself incorrectly position on the reading frame, and the context in which an out of frame stop codon can regulate these errors, is somewhat less clear. We consider three models to this effect. First, the +1 stop codon may abort translation immediately if the ribosome slips following initiation, preventing the synthesis of a faulty protein and allowing ultrarapid recycling of ribosomes which are often rate-limiting (Shah et al. 2013; Subramaniam et al. 2014) (translation termination). Alternatively, the stop codon might provide a regulatory signal to increase the fidelity of the ribosome locating the correct initiation site (frameshift “stop and adjust”). It is reasonable to suppose that a slightly misaligned ribosome could read TGA as stop, blocking translation, realigning the ribosome on the correct start site whilst still in the presence of initiation factors. Finally, the +1 TGA may prevent read-through following the translation of an upstream gene (read-through termination), although there may well be many alternative sites for an out of frame stop to determine the fate of frameshifted translation.

We find evidence against the last of these models (see supplementary result 4, Supplementary Material online). Regarding the “stop and adjust” model, this may be configured more generally in a context of start site recognition mechanisms. This model would concur with our observations that fourth site *A* content is associated with an absence of SD sequences or leader genes, both of which are implicated in start codon recognition. Yamamoto et al. (2016) propose that bacterial 70 S ribosomes have the ability to scan the mRNA and the presence of a SD sequence provides an important signal for selection of the correct start codon by allowing the fMet-tRNA to fix the ribosome at the canonical start codon. In its absence, the ribosome is not fixed and can continue to scan the mRNA. Our results in supplementary results 1 and 2, Supplementary Material online, are consistent with fourth site regulation of initiation by assistance in identifying and positioning the ribosome correctly at the start codon when lacking SD sequences and are suggestive of a direct involvement of the fourth site in the dynamics of translation initiation and start codon selection.

The identity of the start codon has also been shown to determine translation efficiency (O'Donnell and Janssen 2001; Osterman et al. 2013; Panicker et al. 2015; Hecht et al. 2017). We proposed two hypotheses that may implicate the start codon with fourth site *A* usage, either contributing to mRNA-ribosome stability for the more efficient start codons, or preventing the ribosomes from dissociating from weaker start codons. We find the *A* enrichment at the fourth site strongest for GTG, followed by ATG and TGT (supplementary result 5, Supplementary Material online) suggesting that the weakest binding initiator has weakest enrichment. Both Panicker et al. (2015) and Osterman et al. (2013) report GTG is the more efficient initiator. The increased enrichment at the more efficient start codons again implicates the fourth site in increasing initiation efficiency, although the evidence is not definitive. Interestingly, stop codons in 5′ leading regions allow termination of translation events that initiate before the ribosome reaches the correct start codon, increasing protein synthesis efficiency (Seligmann 2007). It is possible that the fourth site acts as a final checkpoint against these events, allowing recalibration or reinitiation of the ribosome at the correct initiation site. Such events may occur as increases in the number of alternative start codons in the 5′ region has a measurable increase on protein activity (Seligmann 2007). The evolution of 5′ stop codons to complement the use of these upstream start codons can provide stringent regulation of the ribosome initiation from the correct initiation site, where fourth site *A* can provide site-specific definition of the correct site.

Our results implicate involvement fourth site *A* in translation initiation and are consistent with in ensuring correct start codon selection. Assuming TGA to be an ancestral stop codon, the reduced enrichment for genomes not using the TGA stop suggest this control is functionally related to the presence of the stop codon. Upon losing the TGA stop, selection to maintain this enrichment was reduced and enrichment weakened to levels required for RNA stability.

### *A*_4_ Enrichment Observed in Archaea but Not in Eukaryotes Is Suggestive of Interactions Specific to the Prokaryotic Ribosome

One curiosity concerning fourth site usage is that different patterns are observed in nuclear eukaryotic genes. We find that *A*_4_ enrichment ratios are significantly enriched >1 among archaea genomes (73/77, 94.81%), however we find no evidence for fourth site enrichment specific to *A* within eukaryotes (supplementary result 6, Supplementary Material online). As methionine removal is largely the same in the two taxa, a peptide-based argument seems unable to explain our observations. Furthermore, many human and plant genes tend instead to have GC rich terminal ends (Niimura et al. 2003). One notable distinction between the two is the ribosome. If frameshifting or start site recognition mechanisms differ between the 16S rRNA and 18S rRNA then we might expect differences between the taxa, even though TGA is a stop in almost all taxa. Notably the fourth site *A* enrichment observed in archaea, in which initiation resembles that of bacteria and utilizes 16S rRNA, provides a suggestion that the fourth site is a dual coding mechanism functionally linked with the prokaryotic ribosome and initiation mechanics. Given that leaderless mRNAs can be translated between domains (Grill et al. 2000), current leaderless mRNAs may have evolved from ancestral mRNA in which mRNA recognition and initiation the common ancestor occurred via a ribosome-initiation tRNA complex (Moll et al. 2002).

The strength of *A* bias in both bacteria and archaea, but lacking from eukaryotes, suggests the increased initiation complexity in eukaryotes (Asano 2014) may have allowed relaxed selection on ancestral fourth site *A*, given there are stringent alternative mechanisms for locating the correct start codon. The recruitment of ribosomes to eukaryotic mRNA and subsequent start codon identification requires a combination of eukaryotic initiation factors (eIFs) (Jackson et al. 2010; Shatsky et al. 2014) and further binding proteins, when only three initiation factors are found in bacteria (Laursen et al. 2005). Some bacterial leaderless genes do not require the presence of ribosomal proteins S1 or S2 (Moll et al. 2002), which are required for the 30 S ribosome pathway, or even the presence of initiation factors (Udagawa et al. 2004). Interactions between initiation factors forming multifactor complexes (MFC) provide stringent ATG recognition (reviewed in Asano 2014). eIF1A, a universally conserved eukaryotic homolog of bacterial eIF1 has evolved both N- and C-terminal domains stimulating recruitment of methionyl initiator tRNA to ATG but preventing and discriminating against non-ATG initiation (Pestova and Kolupaeva 2002; Fekete et al. 2005; Nanda et al. 2009; Saini et al. 2010). In addition, selection for nucleotides in the Kozak sequence (Kozak 1986, 1997), which acts to increase the efficiency of eukaryotic translation initiation, may be stronger than that on the fourth site *A* that would provide a similar regulation signal. Interestingly, *A* is the second most prevalent nucleotide at site 4 in Kozak sequences for eight eukaryotic organisms (Grzegorski et al. 2014) which may reflect ancestral selection on the fourth site for *A* that has now weakened due to selection for nucleotides in the Kozak sequence, but still greater than for other nucleotides. The fidelity afforded to eukaryotic start codon recognition through the combination of initiation factors and initiation signals may explain the differences in enrichment between the domains at the fourth site.

### Unresolved Issues

Although *A* enrichment is significantly greater at the fourth site compared with seventh and tenth sites of neighboring codons, both synonymous and nonsynonymous sites in the 5′ domain demonstrate an *A* enrichment. What is unclear about any RNA stability model is why *A*, and not *T*, is preferred. Localized *T* enrichment should provide a similar destabilizing effect as that of *A*, but *T* is consistently underrepresented in comparison with *A* in the first three codons. One possibility is the preference for *A* over *T* might reflect avoidance of *G*:*U* noncanonical base pairs that allow weak base pairing (Varani and McClain 2000) and could introduce unwanted mRNA stability. Results from archaea (supplementary result 6, Supplementary Material online) suggest that selection for *A*/*T* content in the 5′ domain reducing RNA stability is not limited to bacteria, but is infrequent in eukaryotes. Why are eukaryotes different in 5′ stability requirements?

Eukaryote analyses also raise further unresolved issues. Although *A* enrichment cannot be accounted for solely in terms of selection on the peptide in bacteria, the preference for particular non-*A* starting amino acids (alanine, proline, and *T*-starting serine) that facilitate methionine cleavage, and the avoidance of A starting methionine and isoleucine that do not, indicate a selection pressure for amino acids promoting cleavage. However, preferences for *A*-starting amino acids that promote cleavage (threonine, *A*-starting serine) are heightened. With evidence for methionine aminopeptidase activity and second amino acid specificity in eukaryotes (Giglione et al. 2000; Chen et al. 2002; Xiao et al. 2010), if selection was primarily for facilitative amino acids we should also observe an *A* enrichment in eukaryotes, yet this is not apparent. We do not know why this is.

The regulation of translation involves interactions with RNA binding proteins (RBPs) that influence ribosome binding and translation initiation (Babitzke et al. 2009; Van Assche et al. 2015). These interactions directly modulate ribosome binding, alter the mRNA secondary structures or act as a chaperone for the interactions of other RNA effectors. The most likely hypotheses implicating the fourth site in ribosome blocking interactions is one in which the fourth site acts as part of a binding site to which the RBPs bind, blocking initiation, or one in in which the fourth site is enriched to avoid these interactions. For example, the global regulator CsrA binds optimally to the sequence 5′-RUACARGGAUGU-3′ (Dubey et al. 2005; Schubert et al. 2007). The *B. subtilis trp* RNA binding attenuation protein (TRAP) binds with the *ycbK* putative efflux protein at NAG motifs across the initiation region, one of which may be GAG from sites 3 to 6, directly blocking the 30 S ribosome binding (Yakhnin et al. 2006). In a similar manner, the bacteriophage T4 regA binds near the start codons and interactions with the fourth site when binding to the to the consensus sequence 5′-AAAAUUGUUAUGUAA-3′ (Winter et al. 1987; Brown et al. 1997). Enrichment of fourth site *A* may reflect selection for avoidance of this interaction. For CsrA, the fourth site is the outermost nucleotide in the consensus sequence and we expect binding of this site to be less important and under weaker selection than binding with the 5′ UTR (Dubey et al. 2003; Edwards et al. 2011) and GGA core motif (Schubert et al. 2007). Binding of both TRAP and regA are likely to be organism specific. Whilst we cannot definitively discount selection against interactions with RBPs, it is unlikely to explain the near-universal enrichment we observe and are not investigated further within the scope of this work.

### Future Prospects: Experimental Tests

Our observations provide an avenue for experimental testing. Adopting approaches similar to Napolitano et al. (2016) who mutated *A-*starting arginine codons to the CGT synonym would be especially valuable. Their preliminary data supports the exceptionalism of the fourth site. Notably 12 of 13 recalcitrant mutations, including 1 of 2 at the second codon, were in mRNA terminal domains highlighting the importance not only of the terminal domains, but the second codon in particular. Further targeted efforts to resolve the mechanistic basis for this would be valuable. A comparative analysis in both genomes that do and don’t employ TGA as a stop would be especially valuable.

## Materials and Methods

### General

*R* version 3.2.3 (R Core Team 2015) was used for data plotting and statistical analyses. All further scripting was conducted using custom scripts in Python 2.7.10 and Python 3.6.1 (https://www.python.org/) with the Biopython 1.66 package (Cock et al. 2009) and Tcl (http://www.tcl.tk/). Scripts can be found at https://github.com/la466/fourth_site.git. For statistical analyses, N denotes the number of genomes used and means are given with one standard deviation.

### Genome Downloads

Genome sequences of 3,731 bacterial genomes were downloaded from the European Molecular Biology Laboratory (EBML) database (http://www.ebi.ac.uk/Tools/dbfetch/emblfetch? db=embl, last accessed 12th January 2016). Genomes were filtered to include one genome per genus to control for phylogenetic nonindependence (additional genomes of that genus were discounted) larger than 500,000 base pairs leaving 651 genomes. Of these, 646 used translation table 11 and 5 translation table 4. CDS from 205 archaea genomes were downloaded from EMBL (accessed 27th October 2016) and subject to filtering leaving sequences from 77 genomes. Eukaryotic CDSs were downloaded from the Ensembl database (Yates et al. 2016) (ftp://ftp.ensembl.org/pub/release-86/fasta/, last accessed 31st October 2016). The analysis was based on CDSs from the following assemblies (Ensembl release 86 unless stated): *H. sapiens* (GRCh38.p7), *S. cerevisiae* (R64-1-1), *D. melanogaster* (BDGP6), *M. musculus* (GRCm38.p4), *M. mulatta* (Mmul_8.0.1), *O. cuniculus* (OryCun2.0), *B. taurus* (UMD3.1), *G. gallus* (Gallus_gallus-5.0), *C. elegans* (WBcel235), and *A. thaliana* (TAIR10, release 33). 186 protist genomes were downloaded from the Ensembl database (Kersey et al. 2016) (ftp://ftp.ensemblgenomes.org/pub/protists/release-36, last accessed 22nd June 2017).

### CDS Filtering

Every CDS within a genome was filtered, limiting the analysis to genes with a multiple of three nucleotides, containing only canonical *A*, *C*, *T*, or *G* nucleotides, without internal stop codons and those with a stop codon defined by the relevant translation table, either translation table 11 (TAA, TAG, and TGA) or translation table 4 (TAA and TAG) where TGA instead encodes tryptophan. For CDSs passing these filtering criteria, start codon frequencies were calculated. As the frameshift model assumes a NTG start codon, for subsequent analyses only CDSs starting with a NTG start codon (*N* = any nucleotide) were considered. In practice, non-NTG start codons are too rare for meaningful analysis. For eukaryotes, only ATG starts were allowed.

### Calculation of Enrichment Ratios

To account for nucleotide bias within the genome, *A* enrichment ratios were calculated for each genome using

(1) $$A_{n}=\frac{f_{A}\left(n\right)}{F_{A}\left(x\right)},$$

where $A_{n}$* *= *A* ratio at position *n*, $f_{A}(n)$ = proportion of CDSs with *A* at site n and $F_{A}(x)$ = proportion of total codons with *A* in position x, where x corresponds to the intracodon position of n (i.e., if n = 4, x = 1, so we are considering all first codon sites in all CDSs in a genome). *n* can take any value from 1 to the length of the longest gene, although we consider events exclusively at 5′ ends. The same protocol was followed to calculate other nucleotide enrichment ratios and amino acid enrichment ratios.

### Nucleotide Conservation

The variation in nucleotide content in each codon provides a representation of possible exceptionalism and conservation of particular positions. Methods for exploring GC content variation were as in Tang et al. (2010). For each codon position in codons 2–30, the proportion of each nucleotide usage was calculated across all CDSs in each genome. For each genome, the GC proportion for each position was then calculated across all CDSs. Finally, the variance in GC content at each position between genomes provided an overall GC variance.

### Nucleotide Variability between Related Species

A local BLAST database was generated from filtered *E. coli* O157 CDSs using BLAST v2.4.0 (ftp://ftp.ncbi.nlm.nih.gov/blast/executables/blast+/LATEST/). CDSs from *S. flexneri* were queried against the local database. If there was more than one match, the ortholog with the lowest expected value (*E*) and percentage match was chosen.

For each orthologous CDS pair, the nucleotide in the first position in each of the first 11 codons of the *E. coli* sequence was noted and losses from this nucleotide in *S. flexneri* orthologs counted. The proportion of sites changing from each nucleotide in each codon was calculated from the total counts. Comparisons between these species do not assume any evolutionary relationship but simply compares ortholog differences. These variations are conservative as orthologs with the most conserved sequences are chosen. We employ *E. coli* as the focal species and *S. flexneri* as the indicator of the effects of weakened purifying selection, as the strength of selection due to effective population size is considered to be smaller (Hershberg et al. 2007). Thus, we can ask whether a fourth site *A* in *E. coli* is more resilient to change. If so, this would indicate stronger purifying selection on the fourth site.

### Codon Adaptation Index Analysis

Bacterial codon use is often highly nonrandom. Translational selection biases codons toward those rapidly translated tRNAs and with high availability (Ketteler 2012). Highly expressed genes, for which translational errors may prove more costly, typically use a restricted set of preferred codons corresponding to the tRNA repertoire (Rocha 2004) with codon bias strongest in these genes (Higgs and Ran 2008). The Codon Adaptation Index (CAI) (Sharp and Li 1987) is one method of quantifying codon bias. High expression correlates with a high CAI value in several organisms including *E. coli* (dos Reis et al. 2003), and therefore the CAI value is used as a proxy measure for gene expression.

For each genome, a reference set of CDSs for which codon usage was expected to be high was selected to represent the highly expressed genes to include 20 ribosomal genes from *rplA/1* to *rplF/6*, *rplI/9* to *rplU/21*, and *rpsB/2* to *rpsU/21*. Only genomes with annotations for 20 of these genes were considered. CAI indices for each gene in this reference set were calculated using CodonW v1.4.4 (https://sourceforge.net/projects/codonw/) using the “-coa_cu -coa_num 100%” parameters to include all reference CDSs. CAI values for the remaining genes within the genome were calculated using the “-all_indices” parameter, including the fop_file, cai_file, and cbi_file. For *E. coli* O157, CAI values were also calculated using the default indices provided by CodonW and correlated with those calculated from our reference set (ρ = 0.987, *P* < 0.01, Spearman’s rank correlation) to ensure the reference set accurately represented the highly expressed genes.

### Identification of Shine–Dalgarno Sequences

Potential Shine–Dalgarno (SD) sequences were identified using methods described in Starmer et al. (2006). For each genome, the 16 S rRNA genes were located and the 3′ tail isolated from the gene sequence. Tails were scanned for the 5′-GAT-3′ motif located closest to the 3′ end of the rRNA tail. If multiple tails were present, the most frequent was selected. Only tails between 8 and 15 nucleotides were considered.

For each CDS within the genome, the change in free energy ΔG° was calculated using the free_scan script from the free2bind v1.0.1 package (https://sourceforge.net/projects/free2bind/) (Starmer et al. 2006). ΔG° describes the change in free energy required to bring the mRNA strand together with the identified 16S rRNA tail; ΔG° scores less than zero describe a likely interaction. For each CDSs, a 60-nucleotide window centered on the start codon, with *A* of the ATG representing nucleotide 30, was extracted and ΔG° was calculated by aligning the 16S rRNA tail at each position in this window. The position with minimal ΔG° was considered the optimal binding site.

A CDS was considered to have a SD sequence providing the optimal binding site had ΔG° ≤ −3.4535 kcal/mol, derived from the average of free_scan calculations for core motifs 5′-GGAG-3′ (−3.60793 kcal/mol), 5′-GAGG-3′ (−3.60793 kcal/mol) and 5′-AGGA-3′ (−3.144505 kcal/mol) (Ma et al. 2002). Strong binding was defined as ΔG° ≤ −8.4 kcal/mol obtained from binding of the sequence 5′-GGAGGT-3′. Relative gene distances were calculated as the distance of the 5′ *A* in the rRNA sequence flanking the core SD motif relative to the first nucleotide in the start codon, defined as 0. Distances less than one indicate a SD sequence upstream of the start codon.

### Average of Difference Calculations

Preferences or avoidances of each amino acid in the second position was calculated using the average of difference (AOD) score (Tang et al. 2010). AOD scores calculate the difference between the frequencies of an amino acid in the second position compared with the average frequencies compared with all positions in the CDS, using the formula

(2) $${AOD}_{x}=\frac{\sum_{n}\left(f\left(x\right)-F_{x}\right)}{n},$$

where ${AOD}_{x}$ = average of difference score for amino acid x, $f\left(x\right)$ = frequency of amino acid x in the second peptide position, $F_{x}$ = average frequency of amino acid x across all amino acids and n = number of CDSs. Genomes were further categorized equally into low (GC ≤ 44.19%), medium (44.19% < GC ≤ 60.91%) and high (GC < 60.91%) GC to account for underlying biases.

### Distances to Out-of-Frame Stop Codons

For each CDS, removing the first nucleotide from the sequence provided the +1 frameshift sequence. For each codon from codon 2 within the shifted sequence was queried for a suitable stop codon. The position of the first nucleotide of the stop codon in the sequence was defined as the distance to the next stop codon. The same protocol was applied for second and third stop codons.

### Identification of Leader Genes

Leader genes were identified as open reading frames (ORFs) 5′ to the structural CDS using similar methods to Lyubetsky et al. (2014) and Korolev et al. (2016). A CDS was considered providing it was longer than 200 nucleotides, shorter than 10,000 nucleotides and had met previous filtering criteria. For each qualifying CDS, the upstream intergenic region was extracted if >100 nucleotides and <1,400 nucleotides.

Within the intergenic region, all potential ORFs were identified providing they had a regular start codon, were a multiple of three nucleotides, without internal stop codons, had a stop codon defined by the relevant translation table and were longer than six codons. If more than one ORF was identified, the longest ORF was chosen. The algorithm was trained on the *E. coli* O157 genome to identify leader genes as found by Korolev et al. (2016) and subsequently applied to all genomes.

### Multivariate Analysis

A multivariate analysis was conducted using 134 genomes with all available data points. These included: the proportion of CDSs with fourth site *A*, *A* content at sites 6, 7, 9, 10, and 12, the proportion of CDSs with a leader gene the proportion of *A*-starting codons and the genome translation table. Further analysis was conducted on all genomes (*N* = 651) and at the gene level (*N* = 2164911).

## Supplementary Material

Supplementary data are available at *Molecular Biology and Evolution* online.

## Supplementary Material

Supplementary Data
