# Supplementary material for: Adenine Enrichment at the Fourth CDS Residue in Bacterial Genes Is Consistent with Error Proofing for +1 Frameshifts
Source: Mol Biol Evol. 2017 Aug 24;34(12):3064–80. doi: 10.1093/molbev/msx223 (PMC5850271; doi:10.1093/molbev/msx223)
Supplement: Supplementary Data [file msx223_supp.zip › msx223_SuppFigandTables.pdf]

Supplementary Figures and Tables

Supplementary Figures 2
Supplementary Figure S1 2
Supplementary Figure S2 3
Supplementary Figure S3 4
Supplementary Figure S4 6
Supplementary Figure S5 7
Supplementary Tables 8
Supplementary Table 1: Percentage of total coding sequences from all 651 genomes utilising each start codon. 8

Supplementary Figures

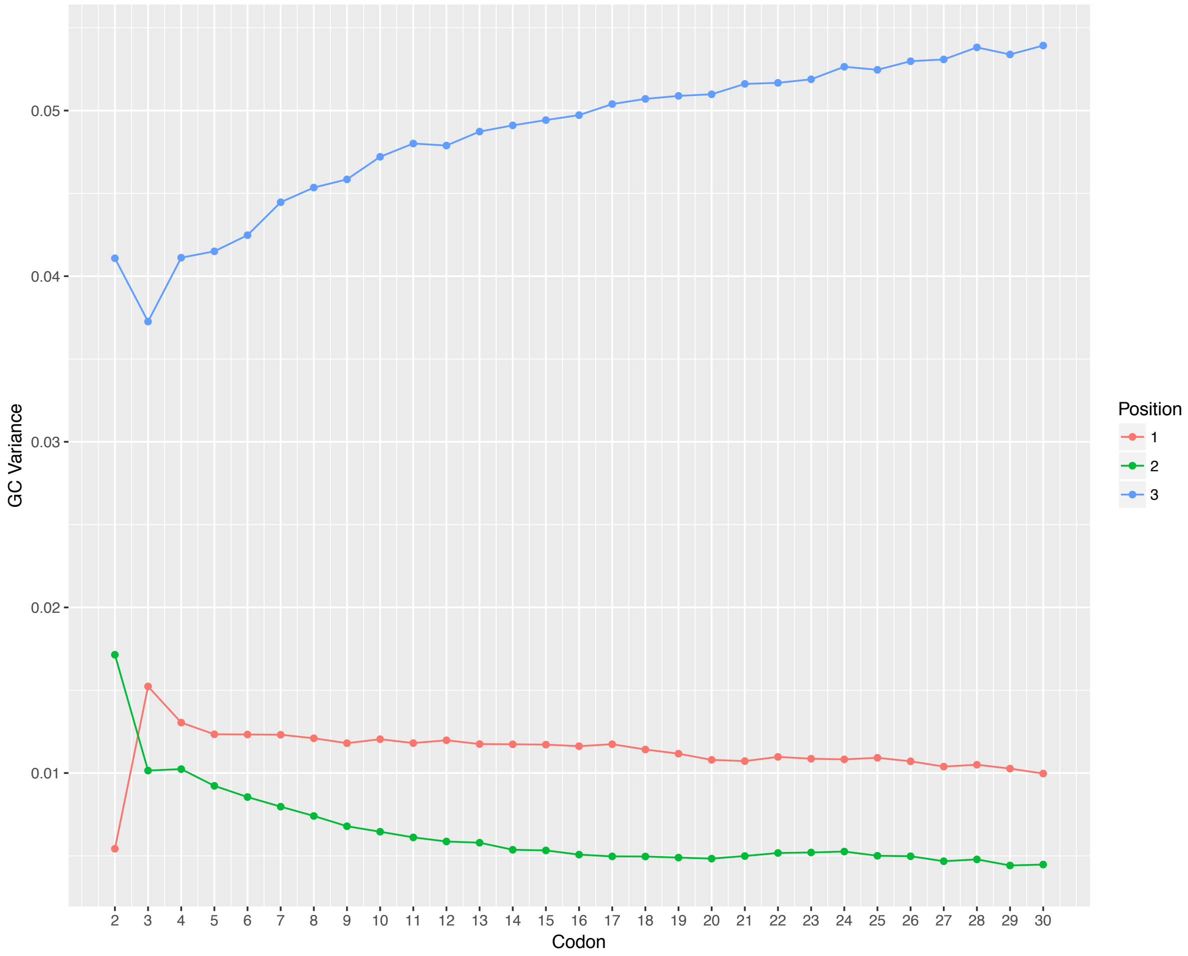

Supplementary Figure S1

The variance in GC content across all genomes for each position in codons 2-30 varies considerably. For each codon except codon 2, GC variance is lowest for the second position. Typically the second codon position is considered to evolve the least quickly (Bofkin and Goldman 2007) and be under greater selection as it is the most functionally constrained (a nucleotide change will bring about a non-synonymous change in codon sequence). However the first position of the second codon (the coding sequence fourth site) exhibits the least GC variance.

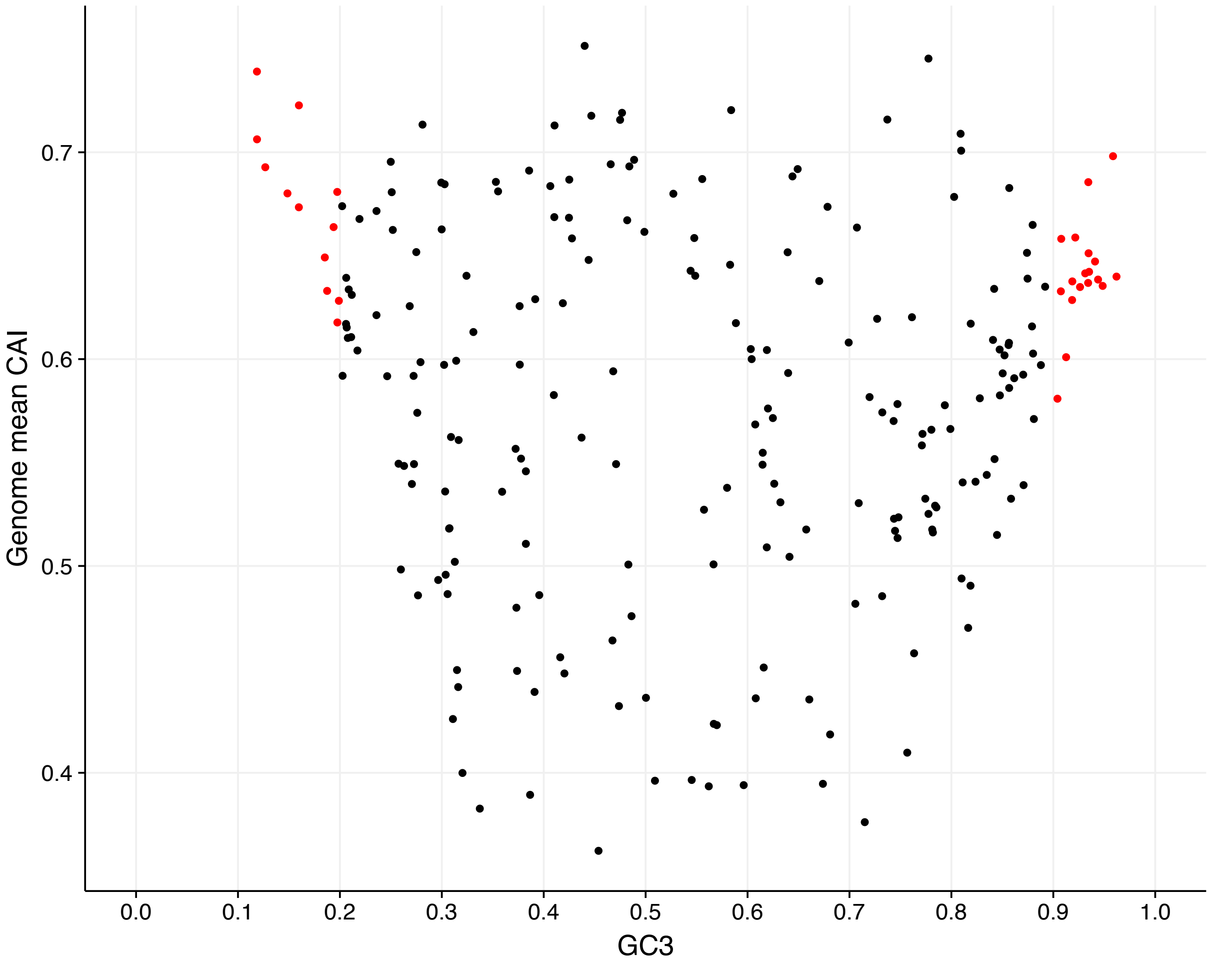

Supplementary Figure S2

The mean of genome Codon Adaptation Index (CAI) values are severely restricted by the genome GC content. Mean CAI values for genomes with extreme GC values (red) are increased indicating many coding sequences are using a set of nearly optimal codons corresponding to those used in the highly expressed genes. Genomes with less restrictive GC content have a greater repertoire of codons and therefore flexibility to use codons that do not correspond to the readily available tRNAs required by highly expressed genes.

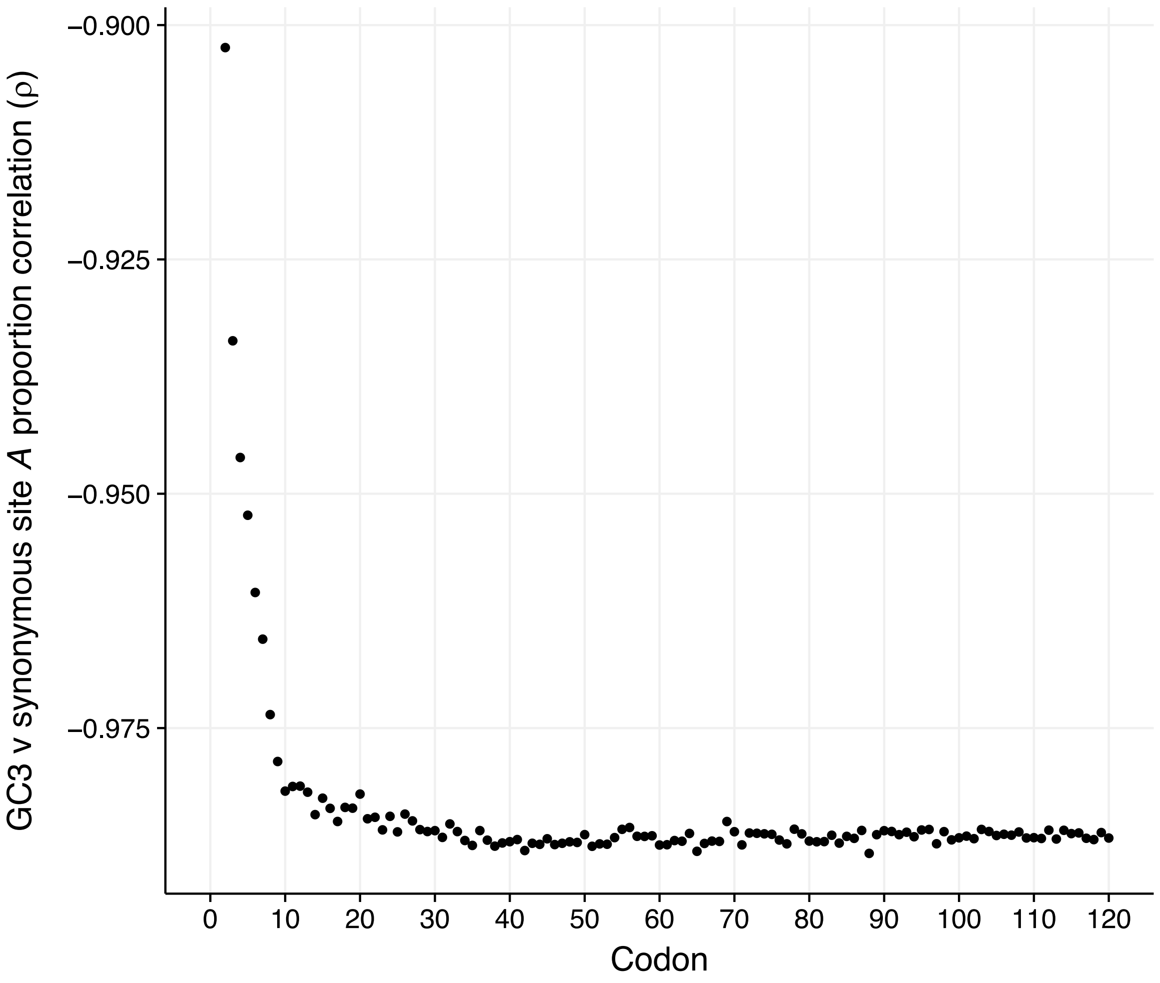

Supplementary Figure S3

Correlations between GC3 content and the proportion of *A* in the synonymous site for codons 2-120. The influence of genome GC content increases moving further from the start codon suggesting codons in the 5’ mRNA domain are under less constraint from genome GC content when determining nucleotide composition.

A

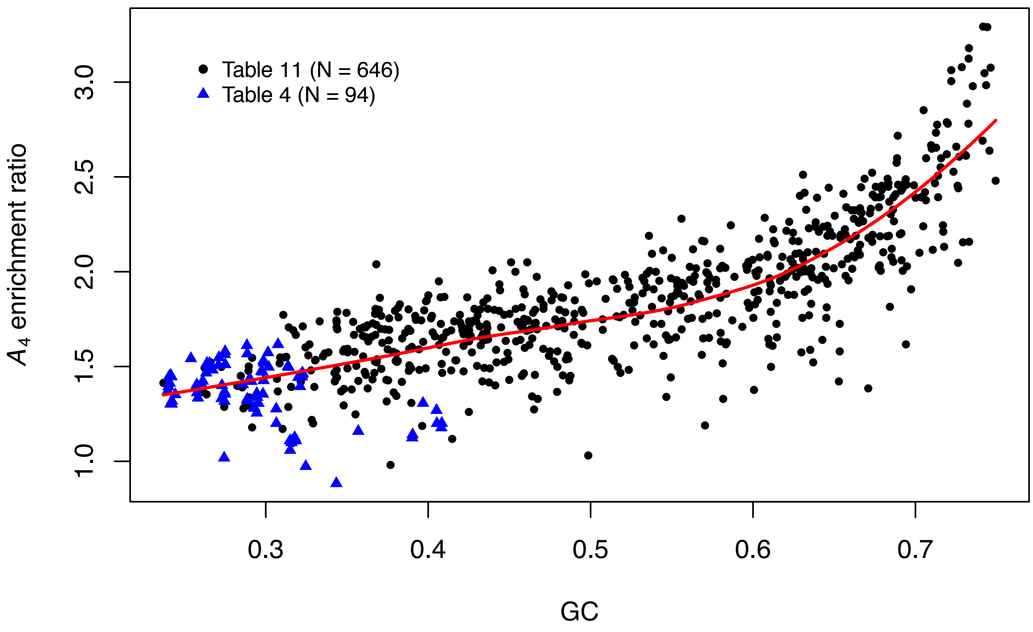

Table 11 (N = 646)
Table 4 (N = 94)
A_4 enrichment ratio
1.0
1.5
2.0
2.5
3.0
0.3
0.4
0.5
0.6
0.7
GC

B

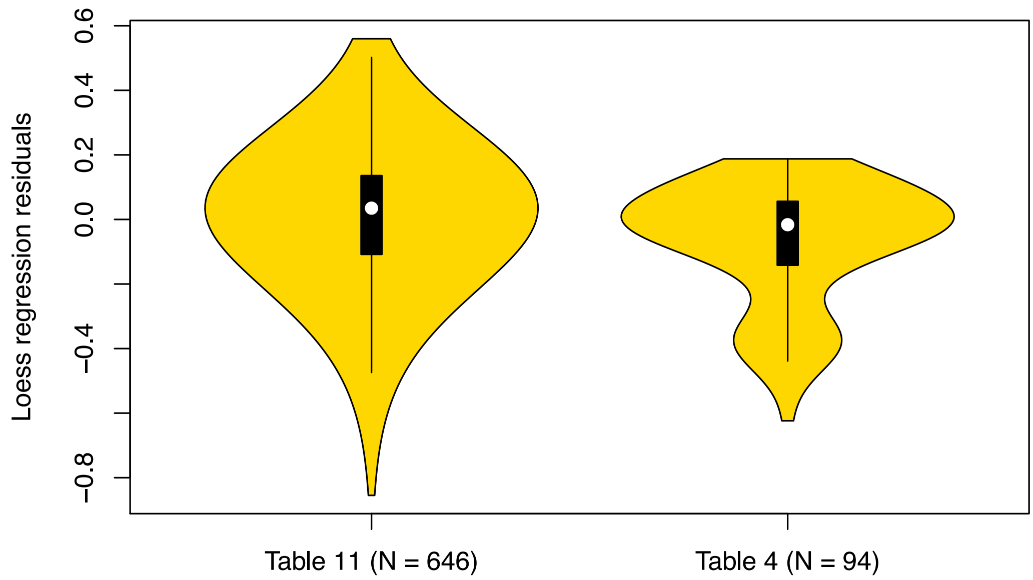

Loess regression residuals
−0.8
−0.4
0.0
0.2
0.4
0.6
Table 11 (N = 646)
Table 4 (N = 94)

C

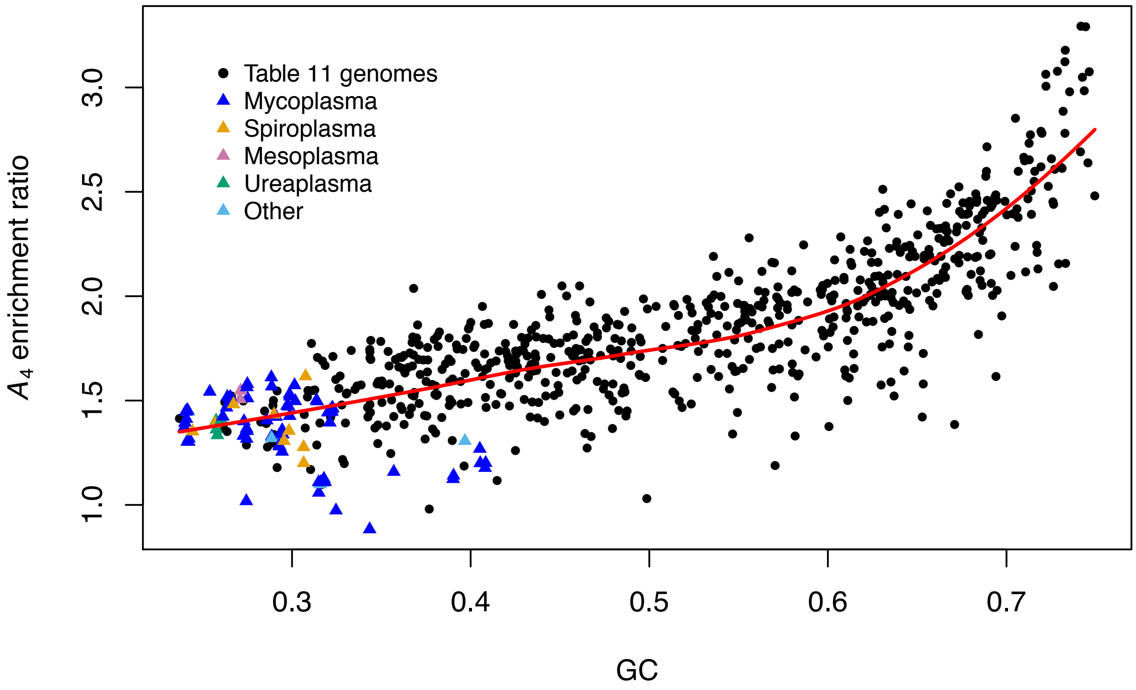

Supplementary Figure S4

A) Loess regression curve of genome GC content against genome A_4 enrichment ratio for both translation tables. Genomes not using a TGA stop (triangle) tend to fall below the loess regression curve. B) Violin plots of the residuals from the loess regression analysis between genome GC content against genome A_4 enrichment ratio demonstrate reduced residuals in genomes not using the TGA stop codon, suggesting *A* enrichment at the fourth site is weaker in these genomes. C) Identical loess regression curve to A, this time differentiating between table 4 genera.

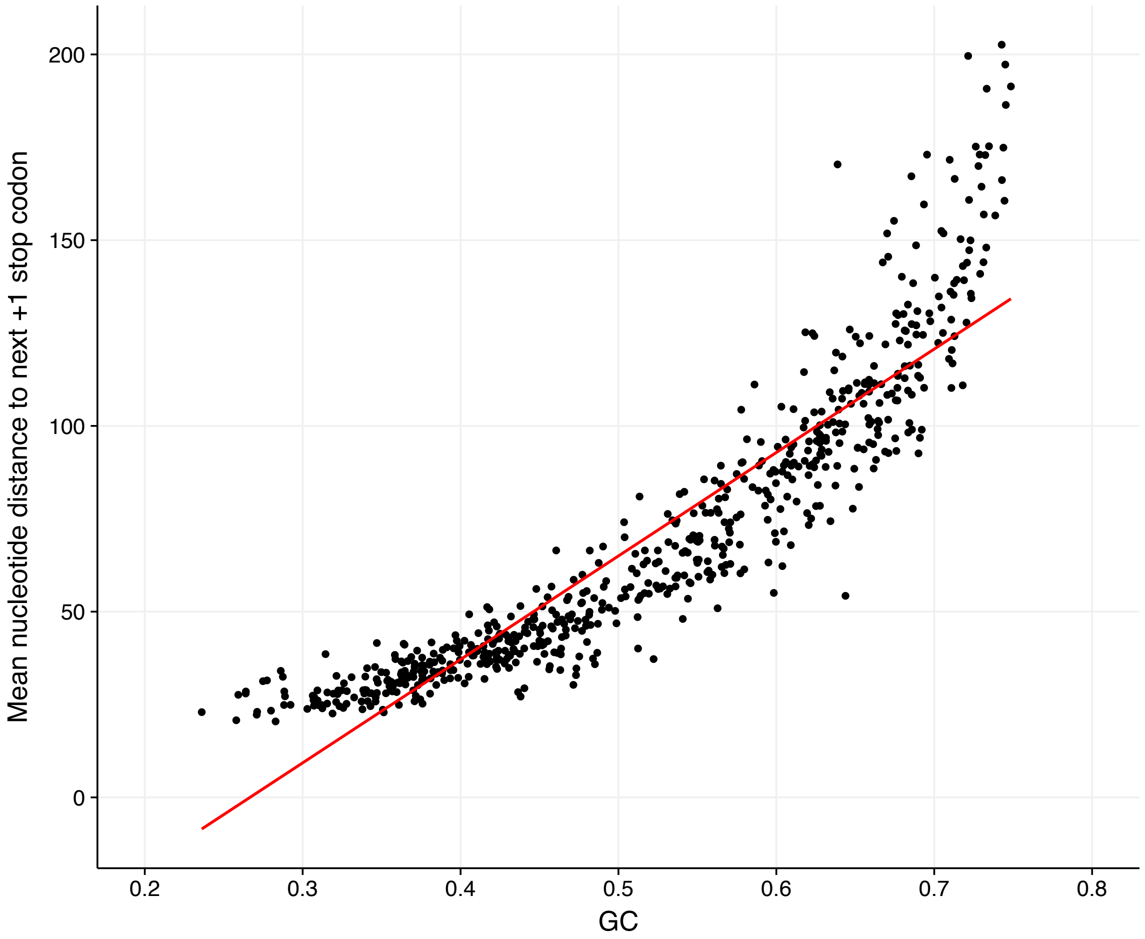

Supplementary Figure S5

The mean distance to the next +1 frameshift stop codon increases with genome GC content. The three stop codons TGA, TAG and TAA are AT-rich and so less likely to be encountered in GC-rich genomes.

Supplementary Tables

Supplementary Table 1: Percentage of total coding sequences from all 651 genomes utilising each start codon.

**A-start codons**	**% use**	**C-start codons**	**% use**	**G-start codons**	**% use**	**T-start codons**	**% use**
AAA	1.29×10^{-5}	CAA	3.22×10^{-6}	GAA	2.76×10^{-6}	TAA	0
AAC	3.22×10^{-6}	CAC	1.84×10^{-6}	GAC	1.84×10^{-6}	TAC	3.22×10^{-6}
AAG	5.51×10^{-6}	CAG	1.84×10^{-6}	GAG	9.19×10^{-7}	TAG	0
AAT	5.97×10^{-6}	CAT	4.59×10^{-7}	GAT	5.05×10^{-6}	TAT	3.67×10^{-6}
ACA	4.59×10^{-6}	CCA	9.19×10^{-7}	GCA	3.67×10^{-6}	TCA	1.38×10^{-6}
ACC	4.59×10^{-7}	CCC	0	GCC	6.43×10^{-6}	TCC	4.59×10^{-7}
ACG	1.38×10^{-6}	CCG	1.84×10^{-6}	GCG	9.19×10^{-7}	TCG	4.59×10^{-7}
ACT	9.19×10^{-7}	CCT	9.19×10^{-7}	GCT	1.38×10^{-6}	TCT	9.19×10^{-7}
AGA	3.67×10^{-6}	CGA	4.59×10^{-7}	GGA	2.76×10^{-6}	TGA	0
AGC	9.19×10^{-7}	CGC	1.38×10^{-6}	GGC	2.30×10^{-6}	TGC	4.59×10^{-7}
AGG	9.19×10^{-7}	CGG	4.59×10^{-7}	GGG	4.59×10^{-7}	TGG	3.67×10^{-6}
AGT	4.59×10^{-7}	CGT	9.19×10^{-7}	GGT	3.22×10^{-6}	TGT	9.19×10^{-7}
ATA	4.01×10^{-4}	CTA	3.67×10^{-6}	GTA	8.27×10^{-6}	TTA	8.73×10^{-6}
ATC	5.24×10^{-4}	CTC	4.13×10^{-6}	GTC	3.22×10^{-6}	TTC	4.13×10^{-6}
ATG	0.8097	CTG	0.0012	GTG	0.1302	TTG	0.0572
ATT	5.25×10^{-4}	CTT	4.59×10^{-6}	GTT	7.35×10^{-6}	TTT	5.05×10^{-6}
