# Supplementary material for: Adenine Enrichment at the Fourth CDS Residue in Bacterial Genes Is Consistent with Error Proofing for +1 Frameshifts
Source: Mol Biol Evol. 2017 Aug 24;34(12):3064–80. doi: 10.1093/molbev/msx223 (PMC5850271; doi:10.1093/molbev/msx223)
Supplement: Supplementary Data [file msx223_supp.zip › msx223_Suppresults.pdf]

Supplementary Results

Supplementary Results 2
Supplementary Result 1 2
Supplementary Result 2 6
Supplementary Result 3 9
Supplementary Result 4 10
Supplementary Result 5 11
Supplementary Result 6 12
References 18

Supplementary Results

Supplementary Result 1

Sequences lacking upstream Shine-Dalgarno sequences have significantly greater fourth site *A* content

The differences in the translation initiation mechanisms between bacteria and eukaryotes suggest distinct pathways have evolved in the translation initiation process. Bacteria, eukaryotes and archaea, whose features resemble those found both in bacteria and eukaryotes, have the ability to translate genes both with and without additional initiation leaders signals in the 5' untranslated region (Tolstrup, et al. 2000; Moll, et al. 2002; Benelli, et al. 2003; Ring, et al. 2007; Akulich, et al. 2016). Leaderless mRNAs are universally translatable (Grill, et al. 2000) between bacteria, archaea and eukaryotes suggesting a common conserved mechanism of initiating leaderless genes. Leaderless initiation is likely the ancestral mechanism (Londei 2005; Zheng, et al. 2011) and in bacteria occurs via strong, more stable interactions with intact 70S ribosomes (Moll, et al. 2002; O'Donnell and Janssen 2002; Moll, et al. 2004; Zuo, et al. 2013) rather than the 30S subunit and is not dependant on ribosomal proteins or initiation factors (Moll, et al. 2004; Udagawa, et al. 2004). Thus, if leaderless genes reflect the ancestral state, why have leader signals evolved?

It is known that features of prokaryotic mRNA 5' untranslated regions (UTR) contribute to the ability and efficiency of translation (Teilhet, et al. 1998; Hayashi, et al. 2016). The conserved Shine-Dalgarno (SD) sequence, 5'-GGAGGT-3' sequence is complementary to the 16S rRNA antiSD sequence (Shine and Dalgarno 1974) and typically located 5-10 nucleotides upstream of the start codon (Chen, et al. 1994) is found in bacteria and archaea but not eukaryotes. Full or partial complementarity of these sequences facilitate binding of the 16S rRNA to the mRNA, correctly positioning the 30S rRNA subunit at the correct start codon (Nakagawa, et al. 2010). The SD sequence is found preferentially in highly expressed genes (Ma, et al. 2002) with mutations in either the SD motif (Velázquez, et al. 1991) or anti-SD motif (Jacob, et al. 1987) reduce protein synthesis levels in *E. coli*, suggesting SD binding provides a precise and critical translation initiation signal. Furthermore in the absence of SD sequences, following initiation the first decoding step at the ribosomal A-site is highly error-prone resulting in significant incorporation of noncognate amino acids (Di Giacco, et al.

2008). When SD sequences are present, there is no evidence of this misincorporation, suggesting SD sequences play a key role in translation initiation accuracy. In the 70S ribosomal scanning model proposed by Yamamoto, et al. (2016), the absence of a SD sequence significantly weakened initiation. They conclude that the SD sequence provides a strong landing signal allowing the fMet-tRNA to fix the 70S ribosome at the cognate AUG. In the absence of a SD sequence the ribosome can continue to scan the mRNA. It is therefore feasible that leaders have evolved to increase initiation accuracy and that other initiation errors may be more frequent in genes lacking an SD. Could the fourth site be acting as an error control mechanism for genes without SD sequences that are likely to be more error prone in selection the correct start codon? If so, we expect a greater use of *A* in those without a SD sequence.

Potential SD sequences were calculated for 399 genomes with suitable 16S rRNA tails. Peaks in the proportion of SD sequences upstream of the start codon (Supplementary Result 1 Figure 1) are consistent with previous work locating SD sequences (Starmer, et al. 2006). The proportion of genome CDSs with SD sequences varies considerably (95.57% in *G. kaustophilus* to 4.87% in *A. pleuropneumoniae*) and we find no correlation between GC3 content and the proportion of genes with a SD sequence (P = 0.155, Spearman's rank correlation).

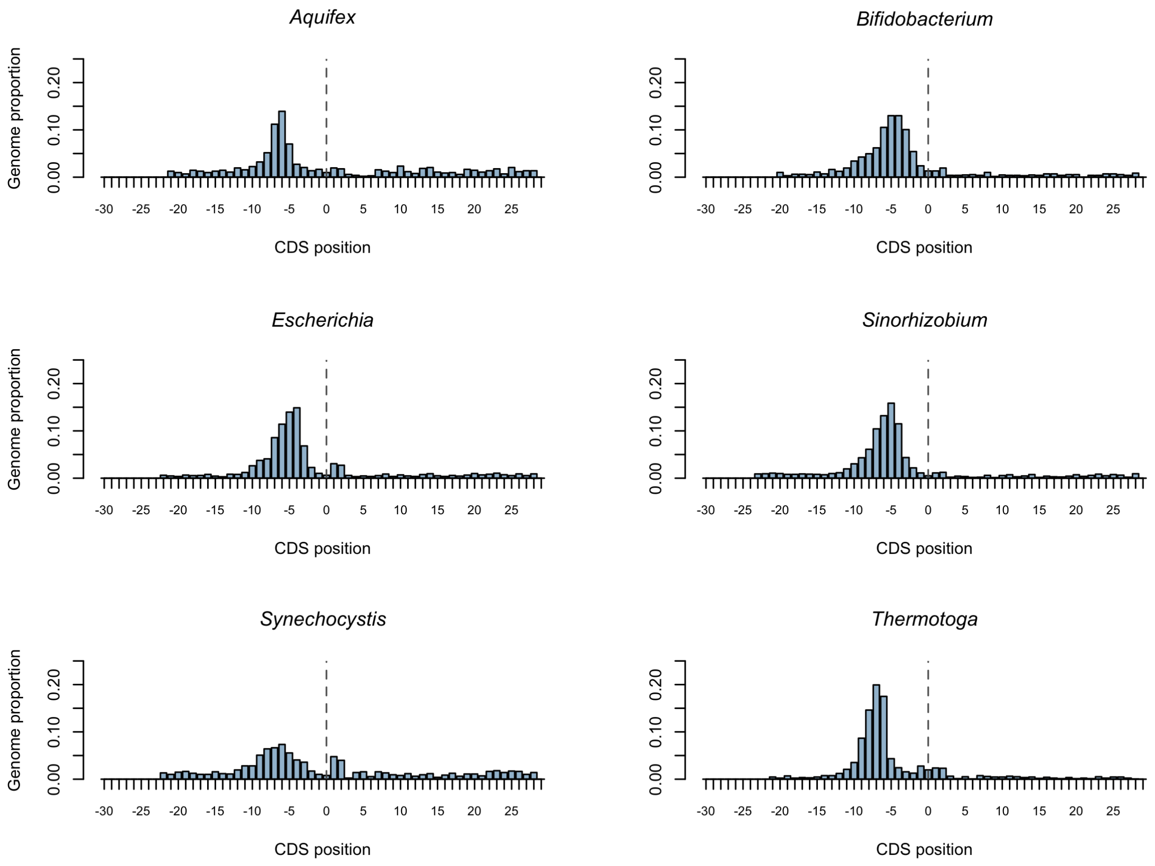

Supplementary Result 1 Figure 1: The location of the strongest binding (ΔG°) between the mRNA and 16S rRNA tail identifies that Shine-Dalgarno (SD) sequences are located 5' of the start codon. Coding sequence position 0 is defined as the first nucleotide of the NTG start codon.

We find the distributions of the proportions of *A* content are extremely similar between CDSs with and without an SD sequence (Supplementary Result 1 Figure 2). The proportion of CDS's with fourth site *A* significantly differs between CDSs with and without a SD (P = 0.002, paired Wilcoxon rank-sum test), with the fourth site *A* proportion marginally greater in genes lacking an SD sequence (mean proportion of CDS with fourth site *A*: with SD: 0.451 ± 0.061; without SD: 0.455 ± 0.065, N = 399). Consistent with fourth site *A* being associated with a lack of SD we find a significant increase in a genome's proportion of genes with fourth site *A* for genes with a weak SD-antiSD interaction compared with strong SD-antiSD interactions (P = 0.013, paired Wilcoxon rank-sum test). As the distance of the SD sequence from the start codon is important (Chen, et al. 1994), we may expect this distance to affect *A* content however we find no difference genome fourth site *A* usage between CDSs with a SD sequence close to the start codon (defined as nearer the start codon than the mean SD

distance) to those with a SD sequence further away (P = 0.638, paired Wilcoxon rank-sum test).

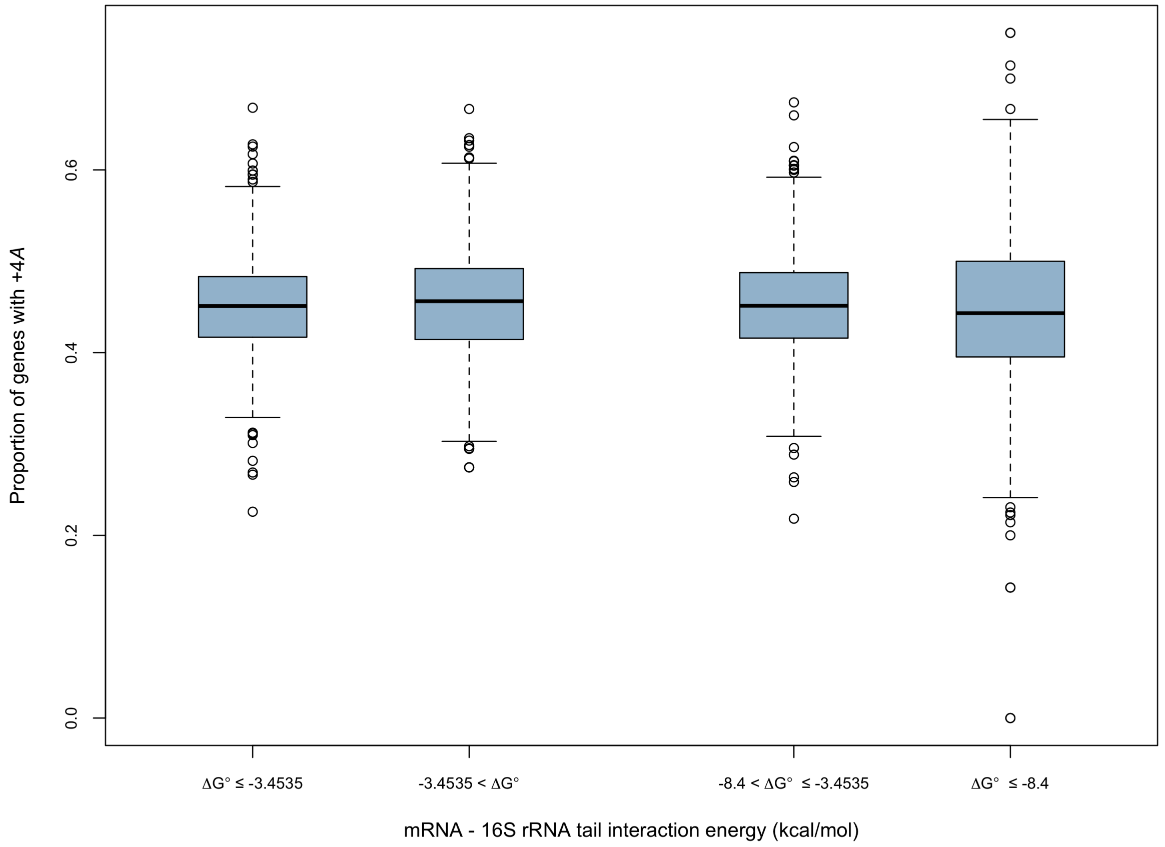

Supplementary Result 1 Figure 2: Distributions of the proportions of genes with +4*A* in the presence of Shine-Dalgarno (SD) sequences (ΔG° ≤ -3.4535 kcal/mol) are similar to those with no SD sequence (ΔG° > -3.4535 kcal/mol). The median proportion of genes with +4*A* of non-SD genes is slightly greater than SD-led genes. The proportion for genes with a strong SD sequence and high complementarity between the 5' mRNA UTR and anti-SD sequence, is more variable (ΔG° ≤ -8.4 kcal/mol) than those with a weak SD (-8.4 < ΔG° ≤ -3.4535 kcal/mol) and has a lower median proportion. These results support the model in which *A* at the fourth site is facilitating translation initiation accuracy in the absence of SD sequences.

Supplementary Result 2

The presence of a leader gene reduces the fourth site *A* prevalence

If fourth site *A* enrichment assists in reducing initiation errors and increases 5' RNA stability, as the data seem to suggest, why then don't all genes use fourth site *A*? Naturally part of the explanation must be mutation-selection equilibrium, which will predict a dynamic equilibrium between mutations removing *A* and selection favouring *A*. The lack of a SD sequence also weakly predicts increased *A* usage. Is there an alternative explanation? Here we demonstrate that the presence of leader genes, as opposed to genes with an addition leader signal in **Supplementary Result 1**, appears to explain some instances of non-fourth site *A*.

Bacteria use a variety of premature termination signals to control gene expression. Genes regulated in this way contain termination signals located in non-protein coding leader genes 5' of the structural mRNA, with up to 10% of operons regulated by a transcription attenuation mechanism (Henkin and Yanofsky 2002) with attenuation signals varying between species and the expressed structural gene. Of particular interest in this study are 5' leader peptides translated prior to CDS translation to situate the ribosome within the vicinity of the CDS (Naville and Gautheret 2010). The influence a leader gene may have on a structural CDS fourth site *A* content is however unknown. High resolution 70S ribosome imaging in the elongation phase indicates approximately 30 nucleotides of the mRNA transcript are encompassed by the ribosome from positions -18 to +12 relative to the current translation site (Demeshkina, et al. 2010). If the distance between the leader gene stop codon and the start codon of a structural CDS is short, is it possible the ribosome simultaneously accommodates both the leader gene and structural CDS, guiding the ribosome to the translation initiation site and facilitating the re-initiation of translation (Korolev, et al. 2016). Leader genes could be described as a 'signpost', helping the ribosome track to the correct start codon and reducing initiation errors.

Potential leader genes were identified as described in the Methods. The specific function of the leader gene was not considered, merely the presence upstream of the CDS. The proportion of CDSs with potential leader genes varies across genomes, from 8.08% in *H. thermophilus* to 76.48% in *T. erythraeum* and is significantly but weakly correlated with GC content (ρ = -0.089, P = 0.023, Spearman's rank correlation). We find a peak in the frequency

of leader genes 10-13 nucleotides upstream from the CDS (Supplementary Result 2 Figure 1) and a reduced peak 3-5 nucleotides upstream from the CDS. Leader genes at these peaks could accommodate the downstream CDS within the ribosome during translation. GC-rich genomes typically have longer CDSs (Xia, et al. 2003) and so these short distances could be attributed to genomes having poor GC content. However, we find a significant negative correlation between GC content and mean distance from leader gene to the CDS (ρ = -0.422, $P < 2.2 \times 10^{-16}$, Spearman rank correlation).

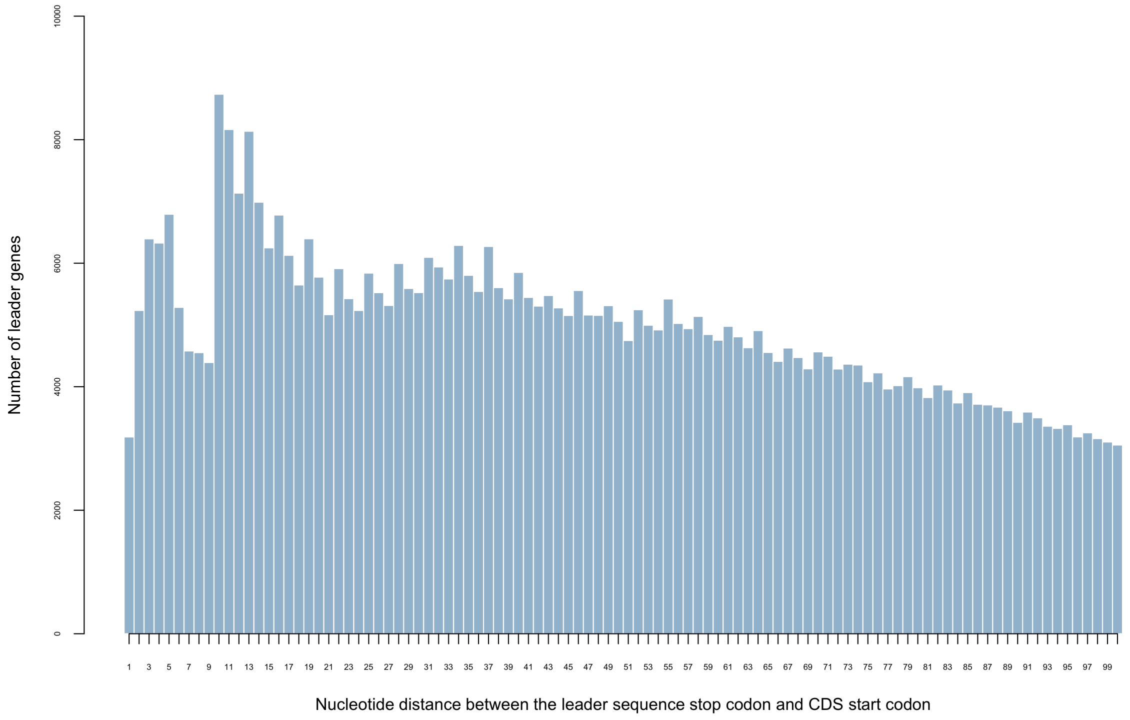

Supplementary Result 2 Figure 1: The number of leader genes at each nucleotide distance to the downstream coding sequence start codon for all genomes. Two peaks of distances are observed at 3-5 and 10-13 nucleotides from the coding sequences.

Does the presence of a leader gene influence fourth site *A* content? Comparing the proportion of fourth site *A* in genes with a leader gene and those without in each genome, we find a significant reduction for those with a preceding leader gene ($P < 2.2 \times 10^{-16}$, paired Wilcoxon rank-sum test; mean *A* proportion for CDSs with a leader = 0.436 ± 0.080 (N = 646), mean *A* proportion for CDSs without leader = 0.480 ± 0.062 (N = 646)). However, we find little variation in the proportion of genes with fourth site *A* as the distance from the CDS increases (Supplementary Result 2 Figure 2), with *A* content not correlated with the nucleotide distance of the leader gene from the CDS (ρ = 0.113, *P* = 0.263, Spearman's rank correlation). It

would seem the presence of a leader gene does influence the *A* content of the fourth site, but is unaffected by the distance of the leader gene from the CDS.

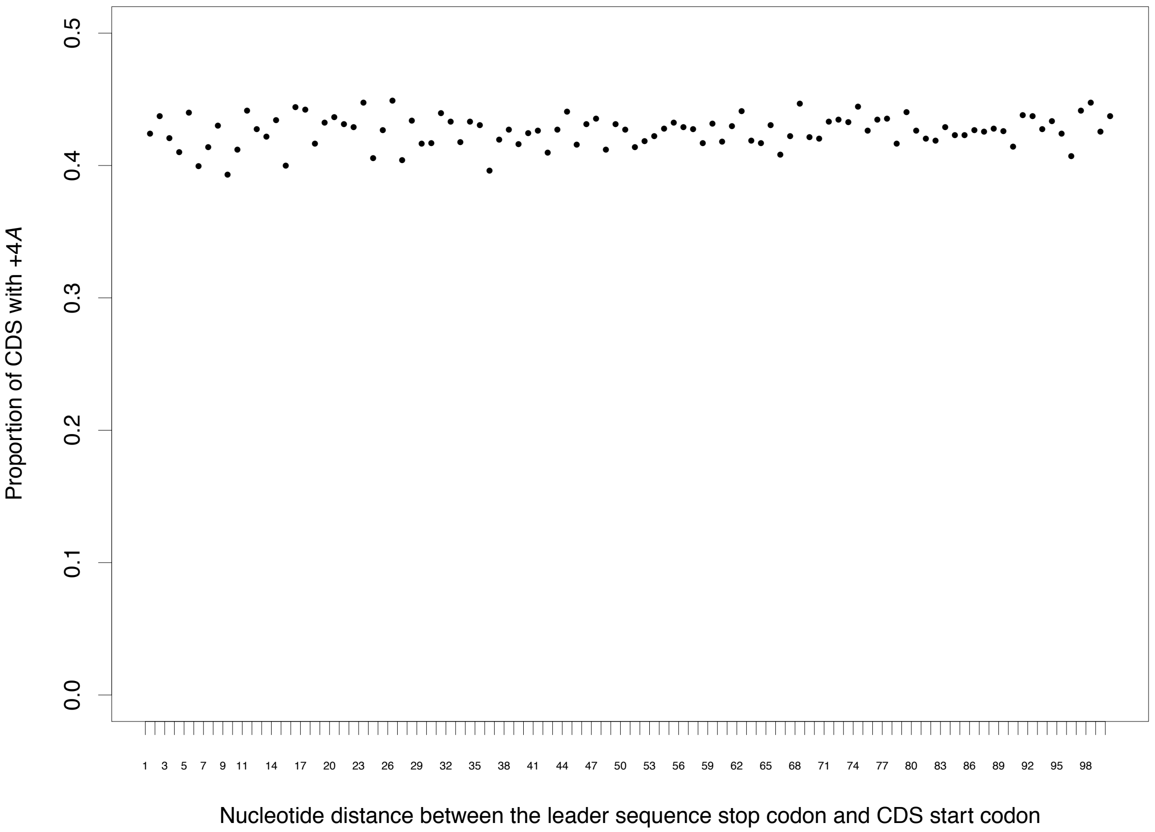

Supplementary Result 2 Figure 2: The proportion of coding sequences with fourth site A in relation to the nucleotide distance of the leader gene from the coding sequence. There is no clear evidence that the distance of the coding sequence from the leader gene has an influence the incorporation of A at the fourth site.

Supplementary Result 3

Multivariate analysis

We have considered each model separately and discussed the implications for fourth site *A* content. May this trend be a combination of selection pressures that determine the ultimate composition of the fourth site? We performed a multivariate analysis predicting the proportions of CDSs with fourth site *A*. As we have measures for two weak predictors (SD and CAI) for a small subset of all genomes, we eliminate these variables. Models using these predictors do not significantly fit the data. Our resulting model (N = 651) explains 54.73% of the variation (adjusted R-squared: 0.5473) with overall significant fit (F-statistic: 197.5 on 4 and 646 df, $P < 2.2 \times 10^{-16}$), with 5' *A* richness ($P < 2 \times 10^{-16}$), proportion of leader genes ($P = 5.45 \times 10^{-11}$) and proportion of *A*-starting codons ($P = 5.80 \times 10^{-8}$) significant predictors. The genome translation table, determining whether a TGA stop is used, is a nearly significant predictor ($P = 0.053$).

We also consider a gene level model in which use of *A* at the fourth site is a binary variable. Under this model, the genome use of *A*-starting codons ($P < 2.2 \times 10^{-16}$), local 5' *A* richness ($P < 6.35 \times 10^{-9}$) and whether nor not the gene has a leader ($P < 2.2 \times 10^{-16}$) are significant predictors. We also find a significant interaction term between the leader gene and 5' *A* richness ($P < 2.2 \times 10^{-16}$). Again the influence of the translation table is nearly significant ($P = 0.055$).

Supplementary Result 4

CDS fourth site *A* acting to prevent a ribosomal start codon readthrough

Mapping has shown that 60-70% of genes in prokaryotes are transcribed as part of an operon (Sorek and Cossart 2010). If the ribosome were to continue to scan the mRNA downstream of a gene, translating multiple sequences from an operon as part of polycistronic transcript, could the presence of an immediate +1 stop codon prevent readthrough of the start codon? The idea of ribosome scanning is not new (Adhin and van Duin 1990; Osterman, et al. 2012). Furthermore, correct initiation is predominantly (Haimov, et al. 2015) accomplished via a ribosomal scanning mechanism in eukaryotes (Kozak 1978; Agarwal and Bafna 1998; Hinnebusch 2014). Under a bacterial scanning model, Yamamoto, et al. (2016) suggest the 70S ribosome does not dissociate following previous CDS translation termination but continues the surrounding sequence for a SD sequence.

If the fourth site prevents readthrough as the ribosome translocates the mRNA between CDSs, we would expect greater *A*-content in the CDSs with an upstream protein-coding CDS on the same strand. Do we find the number of CDSs with +4*A* and an upstream CDS on the same strand greater than expected by chance, given the total CDSs with +4*A* and an upstream CDS? Excluding overlapping genes (ensuring inter-CDS regions allowing scanning, we observe no significant effect of the strand of the upstream CDS on fourth site *A* content ($P \approx 1$, Pearson's cumulative test statistic (χ^2)). This analysis however accounts for genes located on different operons or at distances in which ribosome scanning is unlikely to occur (mean distance between CDS = 1872.64 nucleotides). Restricting the inter-CDS region to 10, 20, 30, 40, 50, 100, 150 or 200 nucleotides did not influence A content ($P \approx 1$, Pearson's cumulative test statistic (χ^2)). Fourth site *A* content is therefore unlikely to be under selection to provide a translocating ribosome assistance in locating the start codon.

Supplementary Result 5

CDSs with less efficient initiation codon TTG demonstrate weakest fourth site *A* enrichment

CDSs with different start codons are translated with different efficiencies (O'Donnell and Janssen 2001; Osterman, et al. 2013; Panicker, et al. 2015; Hecht, et al. 2017). In vitro ribosome binding strength, as estimated from toeprint assays in *E. coli* assays, revealed 30S subunits bound most efficiently to leadered mRNA containing an ATG, followed by GTG and TTG (O'Donnell and Janssen 2001). We hypothesise two ways in which the start codon identity may determine fourth site *A* usage. First, the fourth site may be used more frequently for weaker binding start codons to prevent the ribosome dissociating with the correct initiation site prematurely. Alternatively, the fourth site *A* may be contributing towards the additional strength of binding for ATG start codons by providing an additional interaction between the ribosome and mRNA.

We find CDSs starting GTG (mean A_4 ratio = 2.607 ± 0.688, N = 646) and ATG (mean A_4 ratio = 1.887 ± 0.367, N = 646) demonstrate greater enrichment than TTG (mean A_4 ratio = 1.274 ± 0.319, N = 646), suggesting the weaker start codons are not compensated for with greater *A* content. This is suggestive that fourth site *A* is not assisting the particularly weak start codons. Panicker, et al. (2015) and Osterman, et al. (2013) report that in some cases, GTG is a more efficient initiator of translation. The role of fourth site *A* may be reflected in this increased initiation efficiency, although the evidence is not definitive and the ribosome may attempt to use an alternative start codon. The reduced *A* content in TTG might reflect lower expression and hence lower associated error cost due to an initiation error, as opposed to increasing the efficiency and accuracy of ATG and GTG. The mean CAI varies significantly dependant on the start codon ($P < 0.001$, Kruskal-Wallis rank sum test). Further, CDSs starting TTG have significantly lower CAI than those starting ATG ($P < 0.001$, pairwise Tukey-Kramer test) but not GTG ($P = 0.371$, pairwise Tukey-Kramer test). Thus, the reduced expression of TTG, in particular when compared with GTG, is unlikely to explain the differences in *A* use.

Supplementary Result 6

Fourth site *A* functionality is specific to prokaryotes

Drawing comparisons with other species may provide further insights into the fourth site functionality. For example, is enrichment specific to bacteria or prokaryotes more generally? Do we observe fourth site *A* enrichment in eukaryotes?

Supplementary Result 6.1 - Fourth site enrichment in archaea is comparable to bacteria

Archaea are an interesting domain to investigate evolutionary links between prokaryotes and eukaryotes. Features of archaeal translation initiation resemble those found both in bacteria and eukaryotes. Initiation factors, for example, have close homologues with eukaryote initiation factors (Kyrpides and Woese 1998). Conversely, mRNA structure and mRNA-ribosome recognition via SD interactions with 16S rRNA anitSD motifs resembles initiation consistent with eubacteria (Condò, et al. 1999; Tolstrup, et al. 2000; Slupska, et al. 2001; Sartorius-Neef and Pfeifer 2004). Archaeal genomes also possess a major proportion of CDSs that lack 5′ UTR sequences entirely (Condò, et al. 1999; Chang, et al. 2006). The ability to translate leaderless mRNA's, absent of SD sequences, and those with a SD sequence suggest that two distinct translational mechanisms exist in archaea (Tolstrup, et al. 2000; Benelli, et al. 2003; Ring, et al. 2007). The unique archaeal initiation dynamics can therefore provide insights into fourth site functionality. If the fourth site is important in translation initiation, in particular with ribosome-mRNA interactions, we predict an *A* enrichment similar to that observed in eubacteria.

Replicating previous analyses, we observe significant enrichment of *A* at the fourth site in 73/77 genomes (94.81%) (P < 0.01, Pearson's cumulative test statistic (χ^2), Bonferroni correction). Enrichment in the 5' domain is suggestive of selection for determining RNA stability; synonymous sites each exhibit enrichment (mean A_6 = 1.533 ± 0.684, mean A_9 = 1.425 ± 0.458, mean A_{12} = 1.513 ± 0.540, N = 77) yet are not significantly different (P = 0.587, Kruskal-Wallis rank-sum test; A_6 - A_9: P = 0.780, A_6 - A_{12}: P = 0.940, A_9 - A_{12}: P = 0.570, pairwise Tukey-Kramer tests). However, as with eubacteria, nonsynonymous sites do exhibit localised *A* enrichment (mean A_4 = 1.566 ± 0.405, mean A_7 = 1.187 ± 0.088, mean A_{10} = 1.181 ± 0.106, N = 77) with the fourth site is significantly enriched beyond neighbouring

codons ($P < 2.2 \times 10^{-16}$, Kruskal-Wallis rank-sum test; $A_4 - A_7$: $P = 1.10 \times 10^{-13}$, $A_4 - A_{10}$: $P = 3.40 \times 10^{-14}$, A_7 - A_{10}: $P = 0.980$, pairwise Tukey-Kramer tests). These results suggest archaea are under similar selection pressures at the fourth site.

Supplementary Result 6.2 - Weak *A* enrichment in *S. cerevisiae* may reduce RNA stability but is not observed in the second codon

Does this enrichment extend to eukaryotes? Of interest is *S. cerevisiae*, in which 5' RNA stability is also known to effect expression (Shah, et al. 2013). We therefore also expect an *A* enrichment in the 5' domain for *S. cerevisiae* CDSs. Both nonsynonymous A_7 (1.014) and A_{10} (1.018) ratios and synonymous A_9 (1.073) and A_{12} (1.170) ratios provide evidence of weak selection, yet we do not observe an *A* enrichment in the second codon (A_4 = 0.951, A_6 = 0.966). Interestingly, we find a weak *T* enrichment (T_4 = 1.183, T_6 = 1.110), which may provide the RNA destabilising effect. Notably, we find no evidence of selection specific to fourth site *A*.

Supplementary Result 6.3 - Eukaryotic species exhibit no fourth site enrichment specific to *A*

Is there any evidence of selection consistent with RNA stability or fourth site enrichment in other eukaryotes? We find variable enrichment profiles for codons 2-4 of various eukaryotes (Supplementary Result 6 Figure 1, Supplementary Result Figure 2). *C. elegans, D. melanogaster* and *A. thaliana* each exhibit *T* enrichment at the seventh and tenth sites, whilst *D. melanogaster* and *A. thaliana* exhibit an *A/T* preference in both first and synonymous sites of codons 3 and 4. A reduction in mRNA folding increasing the accessibility of the RNA in the CDS termini in each of these species has previously been documented (Li, Zheng, Ryvkin, et al. 2012; Li, Zheng, Vandivier, et al. 2012; Vandivier, et al. 2013) which these results seemingly confirm. A strong *G/C* bias at each position in the 5' domain of other eukaryotes would suggest that RNA stability selection is not universal. In each eukaryotic species except *C. elegans*, we observe an enrichment of *G* in the fourth site. The Kozak sequence, for which fourth site *G* is an important nucleotide of the canonical GCCRCCAUGG (Kozak 1986, 1997) motif in eukaryotic ribosome binding may explain this enrichment. We also observe *T* enrichment at the fourth site in *C. elegans* and *D. melanogaster* that has previously been described in invertebrates and fungi (Nakagawa, et al.

2008). Whilst *C. elegans* exhibits an enrichment of *A* at the fourth site, this is almost identical to *T* enrichment (A_4 = 1.093, T_4 = 1.090).

This change in enrichment profiles may however reflect the weakened purifying selection in eukaryotes not being able to maintain fourth site *A*. We consider the enrichment in protist genomes, for which effective population sizes are larger and therefore likely to be under stronger purifying selection. As with the previous selected eukaryotes, we find no evidence for specific fourth site *A* enrichment (Supplementary Result 6 Figure 2). The *Paramecium* genome, considered to have a large effective population size (Snoke, et al. 2006), has an A_4 ratio of 1.204. However, 637/646 (98.61%) of bacterial and 68/77 (88.31%) of archaea genomes have a greater A_4 ratio greater than this value. Fourth site ratios for both bacteria ($P < 2.2 \times 10^{-16}$, Wilcoxon rank sum test) and archaea ($P = 1.786 \times 10^{-11}$, Wilcoxon rank sum test) are significantly greater in both cases. The maximum enrichment ratio for the protists is 1.449 for *P. tricornutum*, lower than 586/646 (90.71%) bacterial genomes. Thus, the reduced enrichment is consistent across the eukaryotic domain and unlikely to be due to weakened purifying selection not being able to maintain this enrichment.

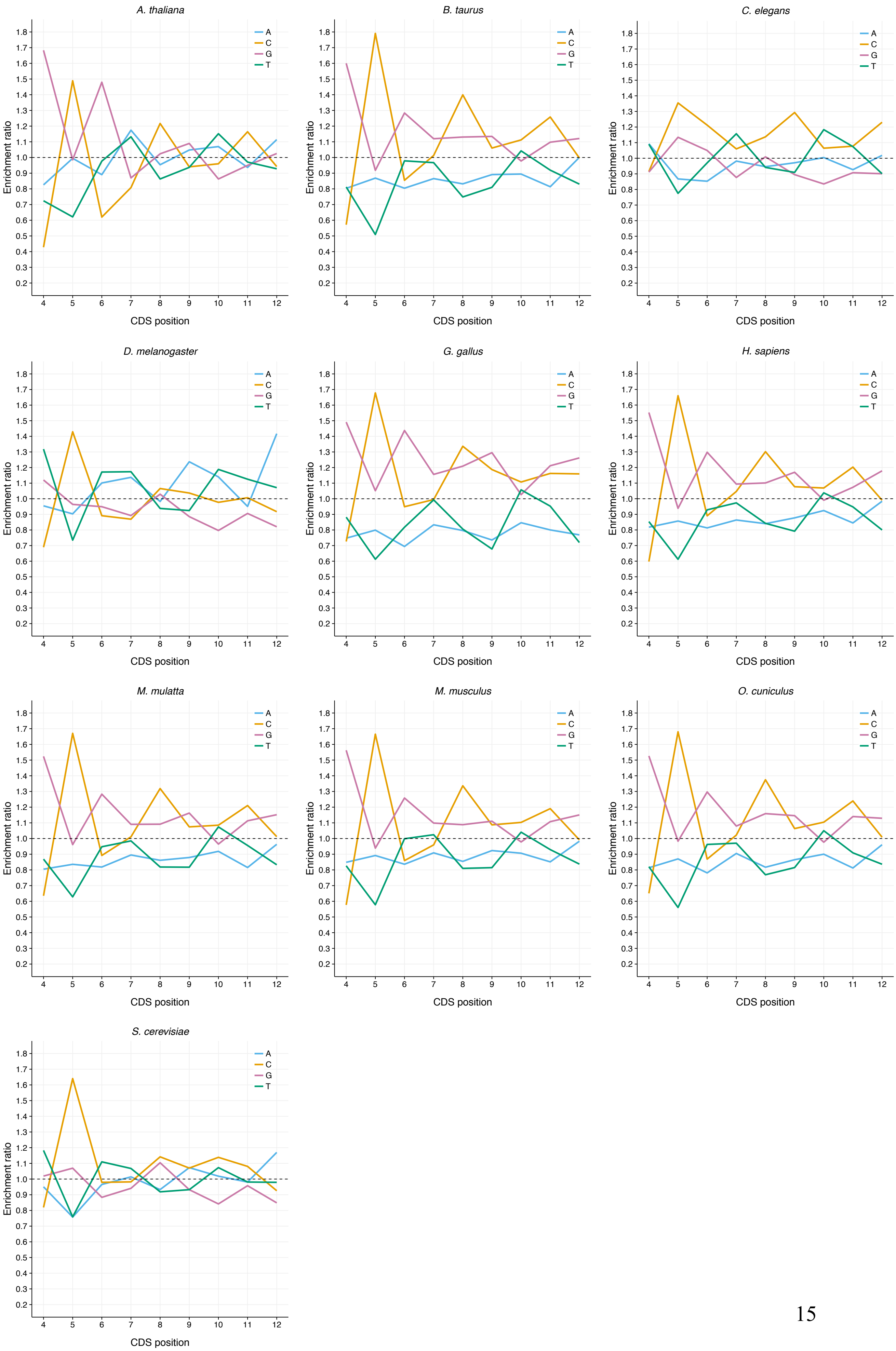

A. thaliana
B. taurus
C. elegans
D. melanogaster
G. gallus
H. sapiens
M. mulatta
M. musculus
O. cuniculus
S. cerevisiae
A
C
G
T
Enrichment ratio
CDS position

Supplementary Result 6 Figure 1: Enrichment ratios in eukaryotes provide no evidence of selection for increased *A* content specific to coding sequence fourth sites. *S. cerevisiae*, *C. elegans*, *D. melanogaster* and *A. thaliana* demonstrate bases towards *A/T* in both synonymous and nonsynonymous sites. A general *G/C* bias is observed in vertebrate CDS, with the fourth site under strong selection for *G* content.

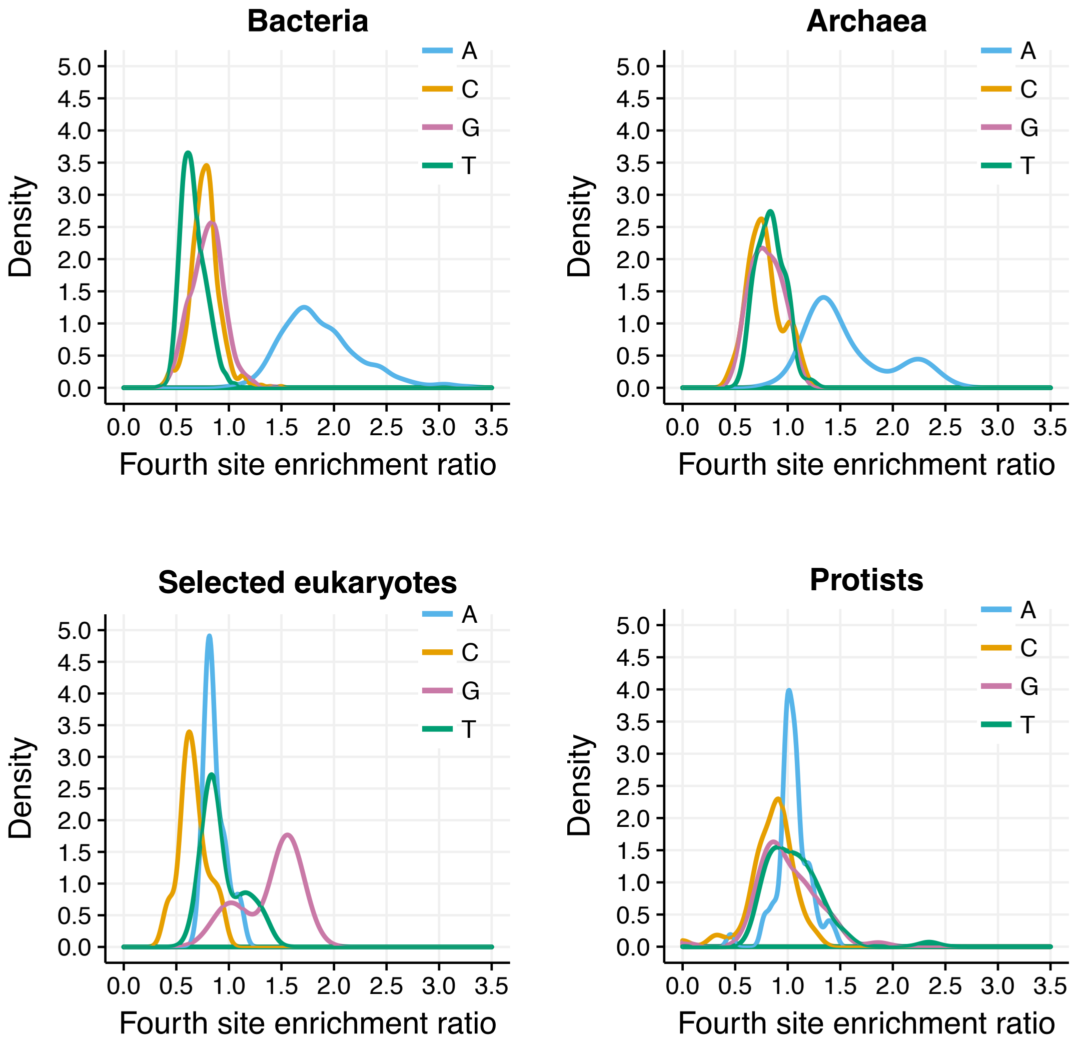

Supplementary Result 6 Figure 2: Enrichment ratios in the selected eukaryotes and protists demonstrate no bias specific to *A* at the fourth site that is observed for both bacteria and archaea. Eukaryotes demonstrate a clear enrichment of *G* at the fourth site, likely to reflect selection for nucleotides within the Kozak sequence.

References

Adhin MR, van Duin J. 1990. Scanning model for translational reinitiation in eubacteria. Journal of Molecular Biology 213:811-818.

Agarwal P, Bafna V. 1998. The ribosome scanning model for translation initiation: implications for gene prediction and full-length cDNA detection. Proc Int Conf Intell Syst Mol Biol 6:2-7.

Akulich KA, Andreev DE, Terenin IM, Smirnova VV, Anisimova AS, Makeeva DS, Arkhipova VI, Stolboushkina EA, Garber MB, Prokofjeva MM, et al. 2016. Four translation initiation pathways employed by the leaderless mRNA in eukaryotes. Sci Rep 6:37905.

Benelli D, Maone E, Londei P. 2003. Two different mechanisms for ribosome/mRNA interaction in archaeal translation initiation. Molecular Microbiology 50:635-643.

Chang B, Halgamuge S, Tang SL. 2006. Analysis of SD sequences in completed microbial genomes: Non-SD-led genes are as common as SD-led genes. Gene 373:90-99.

Chen H, Bjerknes M, Kumar R, Jay E. 1994. Determination of the optimal aligned spacing between the Shine-Dalgarno sequence and the translation initiation codon of Escherichia coli mRNAs. Nucleic Acids Research 22.

Condò I, Ciammaruconi A, Benelli D, Ruggero D, Londei P. 1999. Cis-acting signals controlling translational initiation in the thermophilic archaeon Sulfolobus solfataricus. Molecular Microbiology 34:377-384.

Demeshkina N, Jenner L, Yusupova G, Yusupov M. 2010. Interactions of the ribosome with mRNA and tRNA. Current Opinion in Structural Biology 20:325-332.

Di Giacco V, Márquez V, Qin Y, Pech M, Triana-Alonso FJ, Wilson DN, Nierhaus KH. 2008. Shine–Dalgarno interaction prevents incorporation of noncognate amino acids at the codon following the AUG. Proceedings of the National Academy of Sciences 105:10715-10720.

Grill S, Gualerzi CO, Londei P, Blasi U. 2000. Selective stimulation of translation of leaderless mRNA by initiation factor 2: evolutionary implications for translation. EMBO Journal 19:4101-4110.

Haimov O, Sinvani H, Dikstein R. 2015. Cap-dependent, scanning-free translation initiation mechanisms. Biochimica et Biophysica Acta (BBA) - Gene Regulatory Mechanisms 1849:1313-1318.

Hayashi R, Sugita C, Sugita M. 2016. The 5′ untranslated region of the rbp1 mRNA is required for translation of its mRNA under low temperatures in the cyanobacterium Synechococcus elongatus. Archives of Microbiology:1-8.

Hecht A, Glasgow J, Jaschke PR, Bawazer LA, Munson MS, Cochran JR, Endy D, Salit M. 2017. Measurements of translation initiation from all 64 codons in E. coli. Nucleic Acids Research 45:3615-3626.

Henkin TM, Yanofsky C. 2002. Regulation by transcription attenuation in bacteria: how RNA provides instructions for transcription termination/antitermination decisions. Bioessays 24:700-707.

Hinnebusch AG. 2014. The Scanning Mechanism of Eukaryotic Translation Initiation. Annual Review of Biochemistry 83:779-812.

Jacob WF, Santer M, Dahlberg AE. 1987. A single base change in the Shine-Dalgarno region of 16S rRNA of Escherichia coli affects translation of many proteins. Proceedings of the National Academy of Sciences of the United States of America 84:4757-4761.

Korolev SA, Zverkov OA, Seliverstov AV, Lyubetsky VA. 2016. Ribosome reinitiation at leader peptides increases translation of bacterial proteins. Biology Direct 11:20.

Kozak M. 1978. How do eucaryotic ribosomes select initiation regions in messenger RNA? Cell 15:1109-1123.

Kozak M. 1986. Point mutations define a sequence flanking the AUG initiator codon that modulates translation by eukaryotic ribosomes. Cell 44:283-292.

Kozak M. 1997. Recognition of AUG and alternative initiator codons is augmented by G in position +4 but is not generally affected by the nucleotides in positions +5 and +6. EMBO Journal 16:2482-2492.

Kyrpides NC, Woese CR. 1998. Universally conserved translation initiation factors. Proc Natl Acad Sci U S A 95:224-228.

Li F, Zheng Q, Ryvkin P, Dragomir I, Desai Y, Aiyer S, Valladares O, Yang J, Bambina S, Sabin Leah R, et al. 2012. Global Analysis of RNA Secondary Structure in Two Metazoans. Cell Reports 1:69-82.

Li F, Zheng Q, Vandivier LE, Willmann MR, Chen Y, Gregory BD. 2012. Regulatory Impact of RNA Secondary Structure across the Arabidopsis Transcriptome. The Plant Cell 24:4346-4359.

Londei P. 2005. Evolution of translational initiation: new insights from the archaea⋆. FEMS Microbiology Reviews 29:185-200.

Ma J, Campbell A, Karlin S. 2002. Correlations between Shine-Dalgarno sequences and gene features such as predicted expression levels and operon structures. Journal of Bacteriology 184:5733-5745.

Moll I, Grill S, Gualerzi CO, Bläsi U. 2002. Leaderless mRNAs in bacteria: surprises in ribosomal recruitment and translational control. Molecular Microbiology 43:239-246.

Moll I, Hirokawa G, Kiel MC, Kaji A, Blasi U. 2004. Translation initiation with 70S ribosomes: an alternative pathway for leaderless mRNAs. Nucleic Acids Research 32:3354-3363.

Nakagawa S, Niimura Y, Gojobori T, Tanaka H, Miura K-i. 2008. Diversity of preferred nucleotide sequences around the translation initiation codon in eukaryote genomes. Nucleic Acids Research 36:861-871.

Nakagawa S, Niimura Y, Miura K-i, Gojobori T. 2010. Dynamic evolution of translation initiation mechanisms in prokaryotes. Proceedings of the National Academy of Sciences of the United States of America 107:6382-6387.

Naville M, Gautheret D. 2010. Transcription attenuation in bacteria: theme and variations. Briefings in Functional Genomics 9:178-189.

O'Donnell SM, Janssen GR. 2001. The Initiation Codon Affects Ribosome Binding and Translational Efficiency in Escherichia coli of cI mRNA with or without the 5′ Untranslated Leader. Journal of Bacteriology 183:1277-1283.

O'Donnell SM, Janssen GR. 2002. Leaderless mRNAs bind 70S ribosomes more strongly than 30S ribosomal subunits in Escherichia coli. Journal of Bacteriology 184:6730-6733.

Osterman IA, Evfratov SA, Sergiev PV, Dontsova OA. 2012. Comparison of mRNA features affecting translation initiation and reinitiation. Nucleic Acids Research.

Osterman IA, Evfratov SA, Sergiev PV, Dontsova OA. 2013. Comparison of mRNA features affecting translation initiation and reinitiation. Nucleic Acids Research 41:474-486.

Panicker IS, Browning GF, Markham PF. 2015. The Effect of an Alternate Start Codon on Heterologous Expression of a PhoA Fusion Protein in Mycoplasma gallisepticum. Plos One 10:e0127911.

Ring G, Londei P, Eichler J. 2007. Protein biogenesis in Archaea: addressing translation initiation using an in vitro protein synthesis system for Haloferax volcanii. FEMS Microbiology Letters 270:34-41.

Sartorius-Neef S, Pfeifer F. 2004. In vivo studies on putative Shine–Dalgarno sequences of the halophilic archaeon Halobacterium salinarum. Molecular Microbiology 51:579-588.

Shah P, Ding Y, Niemczyk M, Kudla G, Plotkin JB. 2013. Rate-limiting steps in yeast protein translation. Cell 153:1589-1601.

Shine J, Dalgarno L. 1974. The 3'-terminal sequence of Escherichia coli 16S ribosomal RNA: complementarity to nonsense triplets and ribosome binding sites. Proc Natl Acad Sci U S A 71.

Slupska MM, King AG, Fitz-Gibbon S, Besemer J, Borodovsky M, Miller JH. 2001. Leaderless transcripts of the crenarchaeal hyperthermophile Pyrobaculum aerophilum. Journal of Molecular Biology 309:347-360.

Snoke MS, Berendonk TU, Barth D, Lynch M. 2006. Large Global Effective Population Sizes in Paramecium. Molecular Biology and Evolution 23:2474-2479.

Sorek R, Cossart P. 2010. Prokaryotic transcriptomics: a new view on regulation, physiology and pathogenicity. Nature Reviews: Genetics 11:9-16.

Starmer J, Stomp A, Vouk M, Bitzer D. 2006. Predicting Shine–Dalgarno Sequence Locations Exposes Genome Annotation Errors. PLoS Computational Biology 2:e57.

Teilhet M, Rashid MB, Hawk A, Al-Qahtani A, Mensa-Wilmot K. 1998. Effect of short 5′ UTRs on protein synthesis in two biological kingdoms. Gene 222:91-97.

Tolstrup N, Sensen CW, Garrett RA, Clausen IG. 2000. Two different and highly organized mechanisms of translation initiation in the archaeon Sulfolobus solfataricus. Extremophiles 4:175-179.

Udagawa T, Shimizu Y, Ueda T. 2004. Evidence for the Translation Initiation of Leaderless mRNAs by the Intact 70 S Ribosome without Its Dissociation into Subunits in Eubacteria. Journal of Biological Chemistry 279:8539-8546.

Vandivier LE, Li F, Zheng Q, Willmann MR, Chen Y, Gregory BD. 2013. Arabidopsis mRNA secondary structure correlates with protein function and domains. Plant Signaling & Behavior 8:e24301.

Velázquez L, Camarena L, Reyes JL, Bastarrachea F. 1991. Mutations affecting the Shine-Dalgarno sequences of the untranslated region of the Escherichia coli gltBDF operon. Journal of Bacteriology 173:3261-3264.

Xia X, Xie Z, Li WH. 2003. Effects of GC content and mutational pressure on the lengths of exons and coding sequences. Journal of Molecular Evolution 56:362-370.

Yamamoto H, Wittek D, Gupta R, Qin B, Ueda T, Krause R, Yamamoto K, Albrecht R, Pech M, Nierhaus KH. 2016. 70S-scanning initiation is a novel and frequent initiation mode of ribosomal translation in bacteria. Proceedings of the National Academy of Sciences 113:E1180-E1189.

Zheng X, Hu G-Q, She Z-S, Zhu H. (Zheng2011 co-authors). 2011. Leaderless genes in bacteria: clue to the evolution of translation initiation mechanisms in prokaryotes. BMC Genomics 12:361.

Zuo G, Xu Z, Hao B. 2013. Shigella Strains Are Not Clones of Escherichia coli but Sister Species in the Genus Escherichia. Genomics, Proteomics & Bioinformatics 11:61-65.
